# Meta-analyses of positive psychology interventions: The effects are much smaller than previously reported

**DOI:** 10.1371/journal.pone.0216588

**Published:** 2019-05-29

**Authors:** Carmela A. White, Bob Uttl, Mark D. Holder

**Affiliations:** 1 University of British Columbia, Kelowna, Canada; 2 Mount Royal University, Calgary, Canada; Universitat Wien, AUSTRIA

## Abstract

For at least four decades, researchers have studied the effectiveness of interventions designed to increase well-being. These interventions have become known as positive psychology interventions (PPIs). Two highly cited meta-analyses examined the effectiveness of PPIs on well-being and depression: Sin and Lyubomirsky (2009) and Bolier et al. (2013). Sin and Lyubomirsky reported larger effects of PPIs on well-being (*r* = .29) and depression (*r* = .31) than Bolier et al. reported for subjective well-being (*r* = .17), psychological well-being (*r* = .10), and depression (*r* = .11). A detailed examination of the two meta-analyses reveals that the authors employed different approaches, used different inclusion and exclusion criteria, analyzed different sets of studies, described their methods with insufficient detail to compare them clearly, and did not report or properly account for significant small sample size bias. The first objective of the current study was to reanalyze the studies selected in each of the published meta-analyses, while taking into account small sample size bias. The second objective was to replicate each meta-analysis by extracting relevant effect sizes directly from the primary studies included in the meta-analyses. The present study revealed three key findings: (1) many of the primary studies used a small sample size; (2) small sample size bias was pronounced in many of the analyses; and (3) when small sample size bias was taken into account, the effect of PPIs on well-being were small but significant (approximately *r* = .10), whereas the effect of PPIs on depression were variable, dependent on outliers, and generally not statistically significant. Future PPI research needs to focus on increasing sample sizes. A future meta-analyses of this research needs to assess cumulative effects from a comprehensive collection of primary studies while being mindful of issues such as small sample size bias.

## Introduction

Mental health has often been conceptualized as the absence of negative symptomatology [1]. Traditionally, research and intervention efforts in psychology have reflected this conceptualization by focusing primarily on deficits, disease and dysfunction. Although this focus has been invaluable to psychology, the expanding field of positive psychology offers a complementary approach by focusing on understanding and increasing well-being, defined by Ryan and Deci [2] as “optimal psychological functioning and experience” (p. 1), and the components of well-being including strengths, life satisfaction, happiness, and positive behaviours [3,4]. Together, the traditional approach to psychology along with positive psychology, provide a well-balanced understanding of humanity [4] that is consistent with the World Health Organization’s view that “Health is a state of complete physical, mental, and social well-being and not merely the absence of disease or infirmity.” [5].

Seligman [6] identified five essential factors of well-being: Positive emotions, Engagement, Relationships, Meaning, and Accomplishment (PERMA). More specifically, well-being is made up of two similar, yet distinct components: subjective well-being and psychological well-being. Subjective well-being (SWB), also referred to as hedonic perspective of well-being, is the emotional and cognitive interpretation of the quality of one's life, and is often assessed by examining one’s happiness, affect, and satisfaction with life [7,8]. Psychological well-being (PWB), also referred to as a eudaimonic perspective of well-being, includes positive relations, personal maturity, growth, and independence [9]. PWB reflects a broader, more multidimensional construct than SWB. Ryff developed a model of PWB with six dimensions: (1) Self acceptance (viewing oneself positively); (2) Positive relations with others (the ability to be empathetic and connect with others in more than superficial ways); (3) Autonomy (self-motivation and independence); (4) Environmental mastery (the ability and maturity to control and choose environments that are most appropriate); (5) Purpose in life (a sense of belonging, significance, and chosen direction); and (6) Personal growth (continuously seeking growth and optimal functioning). Both components of well-being have led researchers to different hypotheses and interests, continually providing both similar and dissimilar findings [2,10]. In sum, well-being is a broad, multidimensional, construct that includes one’s affect, satisfaction with life, happiness, engagement with others, personal growth, and meaning and functioning in life.

Thus, although decreasing or eliminating negative symptomatology is necessary, it is not sufficient to achieve overall well-being. Health-care practitioners and researchers must also focus on prevention and intervention strategies that create, build upon, and foster well-being. Positive psychology interventions (PPIs) should be used to supplement approaches that address poor health. Rather than focusing directly on decreasing negative symptomatology, PPIs aim to increase positive affect, meaning in life, and engagement [1]. For healthy populations, the aim is to bring clients from a ‘languishing’ state of being to a ‘flourishing’ state of being [11]. For subclinical and clinical populations, the goals are to significantly reduce negative symptomatology *and* increase well-being [12]. PPIs are typically easy to follow, self-administered, and brief.

Fordyce [13] developed the first documented PPI designed to increase happiness. This PPI was comprised of 14 techniques including spending more time with others, enhancing close relationships, thinking positively, admiring and appreciating happiness, and refraining from worrying. More recent and common interventions developed and tested by Seligman, Steen, Park, and Peterson [4] include: (1) *Gratitude visits/letters*—where participants write and deliver a letter of gratitude to someone who has been particularly kind or helpful in the past, but who was never suitably thanked; (2) *Three good things*–each night for one week participants write down three good things that went well each day and identify the reasons these things went well; (3) *You at your best*–participants write a story of when they were at their best, identify their personal strengths that were utilized in the story, and then read this story and review their personal strengths each day for one week; and (4) *Using signature strengths*–participants complete and receive feedback from the character strengths inventory [14], and then use one of their top five character strengths in a different way each day for one week. There are many other similar interventions, such as loving kindness meditation [15], acts of kindness [16], hope therapy [17], optimism exercises [18], mindfulness-based strength practices [19], well-being therapy [20,21], and positive psychotherapy [1].

Sin and Lyubomirsky [22] published the first meta-analysis of the effectiveness of PPIs. In the ten years since its publication, this meta-analysis has been cited nearly 2,000 times, highlighting the interest in the effectiveness of PPIs. Sin and Lyubomirsky's reported that the PPIs had a moderate effect on improving well-being and decreasing depression. For well-being, the meta-analysis revealed a significant effect size of *r* = .29 (equivalent to *d* = .61) based on 49 studies. For decreasing depressive symptomatology, a significant effect size of *r* = .31 (equivalent to *d* = .65) was found based on 25 studies. Four years later, Bolier, Haverman, Westernhof, Riper, Smit, and Bohlmeijer [23] published a second highly cited meta-analysis of the effectiveness of PPIs focusing only on randomized controlled studies. Bolier et al. reported much smaller effects than Sin and Lyubomirsky. Bolier et al.’s meta-analysis revealed a significant effect size of *r* = .17 (*d* = .34) for subjective well-being, *r* = .10 (*d* = .20) for psychological well-being, and *r* = .11 (*d* = .23) for depression. Moreover, after they removed outlier effect sizes, the effect sizes decreased to *r* = .13 (*d* = .26) for subjective well-being, *r* = .08 (*d* = .17) for psychological well-being, and *r* = .09 (*d* = .18) for depression. Notwithstanding the dissimilar findings of the effect sizes of the PPIs, the high citation rates of these two meta-analyses highlight the recent and widespread interest in positive psychology.

Schuller, Kashdan, and Parks [24] recently criticized Bolier et al.’s [23] meta-analysis as unreasonably selective, narrow, and non-comprehensive. They cautioned against drawing any conclusions from Bolier et al’s meta-analysis for at least the following reasons. First, Bolier et al. substantially truncated their search by excluding studies prior to 1998 (“…the start of the positive psychology movement)” (p. 2). This eliminated earlier interventions including the seminal work of Fordyce [13,25]. Second, Bolier et al. only included studies that referenced “positive psychology”. Because of this inclusion criterion, numerous relevant studies (e.g. studies using the “Best Possible Self” intervention) were omitted. Third, Bolier et al. excluded interventions that utilized meditation, mindfulness, forgiveness, and life-review because reviews and meta-analyses had already been conducted for these types of interventions. However, the elimination of a particular type of intervention or blend of interventions from a meta-analysis is an obstacle to determining how effective PPIs are in general. Moreover, meta-analyses restricted to a specific type of PPI makes it impossible to compare the effectiveness of the full range of PPIs. Because of the restrictive inclusion criteria, the estimated effect sizes are relevant to only the particular blend of PPIs retrieved by Bolier et al. [23]. In any case, because the scope of this meta-analysis was restricted, conclusions regarding the effectiveness of PPIs in general, and the effectiveness of many particular types of PPIs are limited.

In contrast to Bolier et al., Sin and Lyubomirsky’s [22] did not constrain their selection of primary studies and because of this, they identified many more relevant studies than Bolier et al. despite that they published their meta-analysis four years earlier. However, it is impossible to assess how comprehensive Sin and Lyubomirsky’s [22] meta-analysis was because the search for primary studies was not adequately described and therefore, not replicable. For example, the search parameters were not sufficiently described and the search strategy included searching whatever was available in Sin and Lyubomirsky's private libraries and gathering studies from their colleagues. The literature search described in Bolier et al. [23] was similarly not replicable. For example, although Bolier at el. [23] listed numerous terms they used in conducting their searches, they did not specify how they combined them when conducting their searches.

A critical review reveals five additional serious methodological issues that were not adequately addressed in either meta-analysis, that undermine their conclusions, and that may help explain the differences in their findings. First, Sin and Lyubomirsky [22] reported only averaged unweighted *r*s as effect size estimates for well-being and depression (see Table 4, p. 478, in Sin & Lyubomirsky). However, these estimates give the same weight to all studies, regardless of sample size, and are widely considered inappropriate [26].

Second, the previous meta-analyses did not describe in sufficient detail how they calculated effect sizes for each primary study. For example, Sin and Lyubomirsky [22] stated that effect sizes were “computed from Cohen’s *d*, *F*, *t*, *p*, or descriptive statistics” (p. 469). Bolier et al. [23] state that they calculated Cohen’s *d* from the post intervention means and standard deviations and, in some instances, “on the basis of pre- post-change score” without giving any further details. This lack of clarity is especially important because the calculation of effect sizes differs depending on study design (e.g., whether the study is a between-subject or within-subject design; [27]). Thus, effect size calculations can produce different results depending on whether the study used a repeated measure design [28]. In repeated measures designs, when effect sizes are calculated from test statistics such as *F*s, and *ts* using usual formulae, the resulting effect sizes can be substantially inflated [29,30].

Third, Sin and Lyubomirsky’s [22] and Bolier et al.’s [23] meta-analyses included articles that were common to both studies. However, we calculated a relatively low correlation between the effect sizes extracted by Sin and Lyubomirsky [22] and Bolier et al. [23], suggesting that the effect sizes were determined differently in the two meta-analyses.

Fourth, an examination of Sin and Lyubomirsky’s [22] Tables 1 and 2 indicated the presence of small sample size bias. Small sample size bias (also called small study bias) occurs when smaller studies (with less precise findings) report larger effects than larger studies (with more precise findings). Small sample size bias is frequently the result of publication bias. It is well established that journals are much more inclined to publish studies with statistically significant findings than studies reporting null effects [31]. Thus, small studies, which typically report much larger effect sizes than larger studies, are more likely to be published. In turn, small sample size bias has become a significant problem in meta-analyses and numerous methods have been developed for identifying and estimating effect sizes in the presence of small sample size bias [27]. Although Sin and Lyubomirsky [22] noted asymmetry in a funnel plot of their data, they did not include the funnel plots in their article. However, relying on the *Fail-safe N*, they argued that even though publication bias may be present, it is “. . .not large enough to render the overall results nonsignificant” (p. 477) [22]. However, *Fail-safe N* method is no longer considered useful in assessing the significance of small sample bias because it considers only statistical significance rather than substantive or practical significance, and it improperly assumes that effect sizes in the unpublished studies are zero [26].

**Table 1:** Effect sizes determined by the current study, for each well-being measure and each study included in Sin and Lyubomirsky (2009) well-being meta-analysis.

Study	Available data	Measure	PPI condition	*N*_t_	*N*_c_	*N*_total_	*r*
Bedard.2003.1	prepost-msds	SF-36-MH	Mindfulness	10	3	13	.69
Burton.2004.1	post-msds	PA-NS	Writing positive experiences	48	42	90	.54
Cheavens.2006.1	prepost-msds	TSHS	Hope therapy	16	16	32	.17
Cheavens.2006.1	prepost-msds	PIL	Hope therapy	16	16	32	.01
Cook.1998.1	prepost-ancovaF	LSI-A	Reminiscence	18	18	36	.35
Davis.2004.1	post-msds	LSI-Z	Life review therapy	7	7	14	.40
Emmons.2003.1	post-msds	PA-NS	Gratitude	65	67	132	.10
Emmons.2003.3	post-anovaF	PA-NS	Gratitude	33	32	65	.27
Emmons.2003.3	post-anovaF	global life appraisals	Gratitude	33	32	65	.42
Emmons.2003.3	post-anovaF	connection with others	Gratitude	33	32	65	.39
Emmons.2003.3	post-tpvalue	PANAS-P-observer	Gratitude	26	26	52	.26
Emmons.2003.3	post-tpvalue	SWLS-observer	Gratitude	26	26	52	.32
Fava.1998.1	prepost-msds	PWB-AU	Well-being therapy	10	10	20	.12
Fava.1998.1	prepost-msds	PWB-EM	Well-being therapy	10	10	20	.20
Fava.1998.1	prepost-msds	PWB-PG	Well-being therapy	10	10	20	.22
Fava.1998.1	prepost-msds	PWB-PR	Well-being therapy	10	10	20	.22
Fava.1998.1	prepost-msds	PWB-PL	Well-being therapy	10	10	20	.01
Fava.1998.1	prepost-msds	PWB-SA	Well-being therapy	10	10	20	.18
Fava.1998.1	prepost-msds	SQ-RLX	Well-being therapy	10	10	20	.24
Fava.1998.1	prepost-msds	SQ-CON	Well-being therapy	10	10	20	.17
Fava.1998.1	prepost-msds	SQ-PHS	Well-being therapy	10	10	20	-.17
Fava.1998.1	prepost-msds	SQ-FRN	Well-being therapy	10	10	20	.54
Fava.2005.1	prepost-msds	PWB-AU	Well-being therapy	8	8	16	.51
Fava.2005.1	prepost-msds	PWB-EM	Well-being therapy	8	8	16	.54
Fava.2005.1	prepost-msds	PWB-PG	Well-being therapy	8	8	16	.63
Fava.2005.1	prepost-msds	PWB-PR	Well-being therapy	8	8	16	.40
Fava.2005.1	prepost-msds	PWB-PL	Well-being therapy	8	8	16	.62
Fava.2005.1	prepost-msds	PWB-SA	Well-being therapy	8	8	16	.58
Fava.2005.1	prepost-msds	SQ-RLX	Well-being therapy	8	8	16	-.33
Fava.2005.1	prepost-msds	SQ-CON	Well-being therapy	8	8	16	-.23
Fava.2005.1	prepost-msds	SQ-PHS	Well-being therapy	8	8	16	-.12
Fava.2005.1	prepost-msds	SQ-FRN	Well-being therapy	8	8	16	-.20
Fordyce.1977.1*	post-msds	HM—scale	Insight program	48	60	108	.20
Fordyce.1977.1*	post-msds	HM—scale	Fundamentals program	44	60	104	.30
Fordyce.1977.1*	post-msds	HM—scale	Activities program	50	60	110	.34
Fordyce.1977.2*	post-msds	HM–scale (in general)	Fundamentals	39	29	68	.43
Fordyce.1977.2*	post-msds	HM–scale (last month)	Fundamentals	39	29	68	.37
Fordyce.1983.4*	post-msds	SDL-AH	Fundamentals	64	39	103	.18
Fordyce.1983.4*	post-msds	SDL-P	Fundamentals	64	39	103	.18
Fordyce.1983.4*	post-msds	SDL-AV	Fundamentals	64	39	103	.19
Fordyce.1983.4*	post-msds	SDL-LS	Fundamentals	64	39	103	.16
Fordyce.1983.4*	post-msds	SDL-TS	Fundamentals	64	39	103	.23
Fordyce.1983.4*	post-msds	HM—scale	Fundamentals	64	39	103	.15
Fordyce.1983.6*	prepost-msds	HM—scale	Fundamentals	14	13	27	.02
Fordyce.1983.6*	prepost-msds	HM—scale	Fundamentals—personality	10	13	23	.04
Fordyce.1983.6*	prepost-msds	HM—scale	Fundamentals—attitudes & values	12	13	25	.08
Fordyce.1983.6*	prepost-msds	HM—scale	Fundamentals—lifestyle	8	13	21	.15
Freedman.1996.1	prepost-msds	HS	Forgiveness	6	6	12	.72
Froh.2008.1*	post-msds	GS (lately)	List of gratitudes	76	65	141	-.08
Froh.2008.1*	post-msds	GS (next week)	List of gratitudes	76	65	141	.08
Froh.2008.1*	post-msds	BMSLSS–residency	List of gratitudes	76	65	141	.13
Froh.2008.1*	post-msds	BMSLSS–school experience	List of gratitudes	76	65	141	.06
Green.2006.1	prepost-msds	SWLS	Solution coaching	23	25	48	.45
Green.2006.1	prepost-msds	PANAS-P	Solution coaching	25	25	50	.39
Green.2006.1	prepost-msds	HTS-C	Solution coaching	25	24	49	.18
Green.2006.1	prepost-msds	PWB-PG	Solution coaching	25	25	50	.13
Green.2006.1	prepost-msds	PWB-EM	Solution coaching	25	25	50	.34
Green.2006.1	prepost-msds	PWB-AU	Solution coaching	25	25	50	.03
Green.2006.1	prepost-msds	PWB-PR	Solution coaching	25	25	50	.35
Green.2006.1	prepost-msds	PWB-PL	Solution coaching	25	25	50	.50
Green.2006.1	prepost-msds	PWB-SA	Solution coaching	25	25	50	.38
Grossman.2007.1*	prepost-msds	QOL-PA	Mindfulness	39	13	52	.33
King.2000.1	post-msds	D&E-P	Positive aspects of trauma	32	23	55	.06
King.2001.1	post-msds	D&E-NP	Best possible self	19	16	35	-.04
King.2001.1	post-msds	D&E-NP	Trauma and best possible self	22	16	38	.25
Kremers.2006.1	prepost-msds	SPFILS	Self-management	46	73	119	.13
Lichter.1980.1	prepost-msds	PHAHB	Discussion of irrational beliefs	10	13	23	.38
Lichter.1980.1	prepost-msds	HAP-AFFECT	Discussion of irrational beliefs	10	13	23	.22
Lichter.1980.1	prepost-msds	DS-S	Discussion of irrational beliefs	10	13	23	.40
Lichter.1980.2	prepost-msds	HAP-AFFECT	Positive feeling statements	25	23	48	.19
Lichter.1980.2	prepost-msds	DS-S	Positive feeling statements	25	23	48	.29
Low.2006.1	post-msds	PMS-P	Positive thoughts	20	16	36	.09
Lyubomirsky.2011.1	prepost-difmsds	UPL+PL+SWLS+SHS	Gratitude	107	101	208	.08
Lyubomirsky.2011.1	prepost-difmsds	UPL+PL+SWLS+SHS	Optimism	111	101	212	.03
MacLeod.2008.1*	prepost-msds	PANAS-P	Goal setting and planning skills	29	35	64	.27
MacLeod.2008.1*	prepost-msds	SWLS	Goal setting and planning skills	29	35	64	.14
MacLeod.2008.2*	prepost-msds	PANAS-P	Goal setting and planning skills	9	11	20	.42
MacLeod.2008.2*	prepost-msds	SWLS	Goal setting and planning skills	9	11	20	.03
Otake.2006.2*	prepost-difmsds	JSHS	Counting kindness	71	48	119	.25
Rashid.2006.1	post-cohend	PPTI-C	Positive psychotherapy	11	11	22	.41
Reed.2006.1	prepost-msds	PWB-EM	Forgiveness therapy–Well-being therapy	10	10	20	.66
Ruini.2006.1*	prepost-msds	PWB-AU	Well-being therapy	57	54	111	-.07
Ruini.2006.1*	prepost-msds	PWB-EM	Well-being therapy	57	54	111	.04
Ruini.2006.1*	prepost-msds	PWB-PG	Well-being therapy	57	54	111	-.13
Ruini.2006.1*	prepost-msds	PWB-PR	Well-being therapy	57	54	111	-.12
Ruini.2006.1*	prepost-msds	PWB-PL	Well-being therapy	57	54	111	-.21
Ruini.2006.1*	prepost-msds	PWB-SA	Well-being therapy	57	54	111	-.17
Ruini.2006.1*	prepost-msds	SQ-RLX	Well-being therapy	57	54	111	.19
Ruini.2006.1*	prepost-msds	SQ-CON	Well-being therapy	57	54	111	.07
Ruini.2006.1*	prepost-msds	SQ-PHS	Well-being therapy	57	54	111	.15
Ruini.2006.1*	prepost-msds	SQ-FRN	Well-being therapy	57	54	111	-.05
Seligman.2004.1	post-cohend	SWLS	Unspecified	102	83	185	.16
Seligman.2005.1	prepost-msds	SHI	Gratitude visit	80	70	150	NA
Seligman.2005.1	prepost-msds	SHI	Three good things	59	70	129	NA
Seligman.2005.1	prepost-msds	SHI	You at your best	68	70	138	NA
Seligman.2005.1	prepost-msds	SHI	Signature strengths in a new way	66	70	136	NA
Seligman.2005.1	prepost-msds	SHI	Identifying signature strengths	68	70	138	NA
Seligman.2006.1	prepost-msds	SWLS	Positive psychotherapy	14	20	34	-.01
Seligman.2006.2	prepost-msds	SWLS	Positive psychotherapy	11	9	20	.23
Seligman.2006.2	prepost-msds	PPTI	Positive psychotherapy	11	9	20	.40
Sheldon.2006.1	prepost-msds	PANAS-P	Gratitude	21	23	44	-.08
Sheldon.2006.1	prepost-msds	PANAS-P	Best possible self	23	23	46	.30
Smith.1995.1*	prepost-difmsds	HM	Personal happiness	17	12	29	.38
Smith.1995.1*	prepost-difmsds	PHI	Personal happiness	17	12	29	.55
Smith.1995.1*	prepost-difmsds	HM	Personal happiness w/ meditation	7	12	19	.48
Smith.1995.1*	prepost-difmsds	PHI	Personal happiness w/ meditation	7	12	19	.58
Spence.2007.1	prepost-msds	SWLS	Professional coaching	20	17	37	.38
Spence.2007.1	prepost-msds	B-PA	Professional coaching	20	17	37	.16
Spence.2007.1	prepost-msds	PWB-AU	Professional coaching	20	17	37	.40
Spence.2007.1	prepost-msds	PWB-EM	Professional coaching	20	17	37	.13
Spence.2007.1	prepost-msds	PWB-PR	Professional coaching	20	17	37	.07
Spence.2007.1	prepost-msds	PWB-PL	Professional coaching	20	17	37	.35
Spence.2007.1	prepost-msds	PWB-PG	Professional coaching	20	17	37	.35
Spence.2007.1	prepost-msds	PWB-SA	Professional coaching	20	17	37	.28
Spence.2007.1	prepost-msds	SWLS	Peer coaching	20	17	37	.38
Spence.2007.1	prepost-msds	B-PA	Peer coaching	20	17	37	.25
Spence.2007.1	prepost-msds	PWB-AU	Peer coaching	20	17	37	.28
Spence.2007.1	prepost-msds	PWB-EM	Peer coaching	20	17	37	.14
Spence.2007.1	prepost-msds	PWB-PR	Peer coaching	20	17	37	.12
Spence.2007.1	prepost-msds	PWB-PL	Peer coaching	20	17	37	.43
Spence.2007.1	prepost-msds	PWB-PG	Peer coaching	20	17	37	.30
Spence.2007.1	prepost-msds	PWB-SA	Peer coaching	20	17	37	.33
Tkach.2005.1	prepost-msds	SHS	Kindness–Same 3, 1/week	10	47	57	-.13
Tkach.2005.1	prepost-msds	FBR-PA	Kindness–Same 3, 1/week	10	47	57	-.31
Tkach.2005.1	prepost-msds	SWLS	Kindness–Same 3, 1/week	10	47	57	-.35
Tkach.2005.1	prepost-msds	PWB-SA	Kindness–Same 3, 1/week	10	47	57	-.19
Tkach.2005.1	prepost-msds	PWB-PR	Kindness–Same 3, 1/week	10	47	57	-.25
Tkach.2005.1	prepost-msds	SHS	Kindness–Same 3, 3/week	13	47	60	-.11
Tkach.2005.1	prepost-msds	FBR-PA	Kindness–Same 3, 3/week	13	47	60	.04
Tkach.2005.1	prepost-msds	SWLS	Kindness–Same 3, 3/week	13	47	60	.02
Tkach.2005.1	prepost-msds	PWB-SA	Kindness–Same 3, 3/week	13	47	60	.15
Tkach.2005.1	prepost-msds	PWB-PR	Kindness–Same 3, 3/week	13	47	60	0
Tkach.2005.1	prepost-msds	SHS	Kindness–Different 3, 3/week	36	47	83	.15
Tkach.2005.1	prepost-msds	FBR-PA	Kindness–Different 3, 3/week	36	47	83	.05
Tkach.2005.1	prepost-msds	SWLS	Kindness–Different 3, 3/week	36	47	83	-.05
Tkach.2005.1	prepost-msds	PWB-SA	Kindness–Different 3, 3/week	36	47	83	.03
Tkach.2005.1	prepost-msds	PWB-PR	Kindness–Different 3, 3/week	36	47	83	-.03
Tkach.2005.1	prepost-msds	SHS	Kindness–Different 9, 9/week	34	47	81	.03
Tkach.2005.1	prepost-msds	FBR-PA	Kindness–Different 9, 9/week	34	47	81	.07
Tkach.2005.1	prepost-msds	SWLS	Kindness–Different 9, 9/week	34	47	81	.09
Tkach.2005.1	prepost-msds	PWB-SA	Kindness–Different 9, 9/week	34	47	81	.17
Tkach.2005.1	prepost-msds	PWB-PR	Kindness–Different 9, 9/week	34	47	81	.14
Tkach.2005.1	prepost-msds	SHS	Kindness–Any 3, 3/week	48	47	95	-.04
Tkach.2005.1	prepost-msds	FBR-PA	Kindness–Any 3, 3/week	48	47	95	.05
Tkach.2005.1	prepost-msds	SWLS	Kindness–Any 3, 3/week	48	47	95	-.14
Tkach.2005.1	prepost-msds	PWB-SA	Kindness–Any 3, 3/week	48	47	95	-.13
Tkach.2005.1	prepost-msds	PWB-PR	Kindness–Any 3, 3/week	48	47	95	-.04
Tkach.2005.1	prepost-msds	SHS	Kindness–Any 9, 9/week	50	47	97	.10
Tkach.2005.1	prepost-msds	FBR-PA	Kindness–Any 9, 9/week	50	47	97	.07
Tkach.2005.1	prepost-msds	SWLS	Kindness–Any 9, 9/week	50	47	97	.03
Tkach.2005.1	prepost-msds	PWB-SA	Kindness–Any 9, 9/week	50	47	97	.14
Tkach.2005.1	prepost-msds	PWB-PR	Kindness–Any 9, 9/week	50	47	97	.16
Wing.2006.1	prepost-msds	SWLS	Positive experience with cue	58	55	113	-.11
Wing.2006.1	prepost-msds	SWLS	Positive experience	62	55	117	-.05
Zautra.2008.1a	prepost-msds	PANAS-P	Mindfulness	41	30	71	.15
Zautra.2008.1b	prepost-msds	PANAS-P	Mindfulness	6	14	20	.09

Note.

* = non-randomized study

PPI = positive psychology intervention; *N*t = treatment sample size; *N*c = control sample size; *N*_total_ = total sample size; prepost-msds = pre and post means and standard deviations; SF-36-MH = Health Survey Mental Health; post-msds = means and standard deviations from post data only; PA-NS = Positive Affect, not specified; TSHS = The State Hope Scale; PIL = Purpose In Life; prepost-ancovaF = Ancova F statistic from pre and post data; LSI-A = Life Satisfaction Index A; LSI-Z = Life Satisfaction Index Z; post-anovaF = anova F statistic from post data only; post-tpvalue = t statistic and p value from post data only; PANAS-P-observer = Positive and Negative Affect Schedule—Positive–Observer; SWLS—observer = Satisfaction with Life Scale–observer; PWB-AU = Ryff's Psychological Well-Being–Autonomy, PWB-EM = Ryff's Scale of Psychological Well-Being—Environmental mastery; PWB-PG = Ryff's Scale of Psychological Well-Being—Personal growth; PWB-PR = Ryff's Scale of Psychological Well-Being—Positive relations; PWB-PL = Ryff's Scale of Psychological Well-Being—Purpose in life; PWB-SA = Ryff's Scale of Psychological Well-Being–Self-acceptance; SQ-RLX = Kellner's Symptom Questionnaire–Relaxation; SQ-CON = Kellner's Symptom Questionnaire–Contentment; SQ-PHS = Kellner's Symptom Questionnaire—Physical well-being; SQ-FRN = Kellner's Symptom Questionnaire–Friendliness; SHI = Steen Happiness Index; HM = Happiness Measure—'in general' scale; SDL-AH = Self Description Inventory—achieved happiness; SDL-P = Self Description Inventory–personality; SDL-AV = Self Description Inventory—attitudes and values; SDL-LS = Self Description Inventory—life style; SDL-TS = Self Description Inventory—total score; HS = Hope Scale; GS (lately) = Global Satisfaction—'past few weeks'; GS (next week) = Global Satisfaction—'next week'; BMSLSS = Brief Multidimensional Student Life Satisfaction Scale—school experience; SWLS = Satisfaction with Life Scale; PANAS-P = Positive and Negative Affect Schedule–Positive; HTS-C = Hope Trait Scale composite; QOL-PA = Quality of Life—positive affect; D&E-P = Diener & Emmons Positive Affect; D&E-NP = Diener & Emmons Net Positive Mood; SPFILS = Social Production Function Index Level Scale; PHAHB = Pro-Happy and Anti-Happy Beliefs; HAP-AFFECT = Happiness—Affectometer 1; DS-S = Domain Satisfaction (Sum); PMS-A = Profile Mood States—positive mood; prepost-difmsds = pre and post mean differences and standard deviations; UPL+PL+SWLS+SHS = unpleasant affect, pleasant affect, SWLS, and SHS combined; JSHS = Japanese Subjective Happiness Scale; post-cohend–Cohen's d from post data only; PPTI-C = Positive Psychotherapy Inventory—Children's Version; PPTI = Positive Psychotherapy Inventory; PHI—Psychap Inventory; SHS = Subjective Happiness Scale; B-PA = Bradburn—Positive Affect; FBR-PA = Feldman-Garret & Russells—Positive Affect.

**Table 2:** Effect sizes determined by the current study, for each depression measure and each study included in Sin and Lyubomirsky (2009) depression meta-analysis.

Study	Available Data	Measure	PPI condition	*N*_t_	*N*_c_	*N*_total_	*r*
Bedard.2003.1	prepost-msds	BDI-II	Mindfulness	10	3	13	.24
Cheavens.2006.1	prepost-msds	CES-D	Hope therapy	16	16	32	.23
Davis.2004.1	post-msds	SZD	Life Review therapy	7	7	14	.81
Fava.1998.1	prepost-msds	CID-DEP	Well-being therapy	10	10	20	.53
Fava.1998.1	prepost-msds	SQ-DEP	Well-being therapy	10	10	20	.04
Fava.2005.1	prepost-msds	CID-DEP	Well-being therapy	8	8	16	.28
Fava.2005.1	prepost-msds	SQ-DEP	Well-being therapy	8	8	16	.22
Fordyce.1983.4*	post-msds	DAC	Fundamentals	64	39	103	.14
Fordyce.1983.6*	prepost-msds	DAC	Fundamentals	14	13	27	.05
Fordyce.1983.6*	prepost-msds	DAC	Fundamentals—personality	10	13	23	0
Fordyce.1983.6*	prepost-msds	DAC	Fundamentals—attitudes	12	13	25	.18
Fordyce.1983.6*	prepost-msds	DAC	Fundamentals—lifestyle	8	13	21	.26
Freedman.1996.1	prepost-msds	BDI	Forgiveness	6	6	12	.52
Grossman.2007.1*	prepost-msds	HADS-D	Mindfulness	39	13	52	.21
Lichter.1980.2	prepost-msds	BDI	Positive feeling statements	25	23	48	.20
Lin.2004.1	prepost-msds	BDI-II	Forgiveness therapy	14	14	28	.66
Reed.2006.1	prepost-msds	BDI-II	Forgiveness therapy	10	10	20	.61
Ruini.2006.1*	prepost-msds	SQ-DEP	Well-being therapy	57	54	111	-.12
Seligman.2004.1	post-cohend	CES-D	Unspecified	102	83	185	-.15
Seligman.2005.1	prepost-msds	CES-D	Gratitude visit	80	70	150	.16
Seligman.2005.1	prepost-msds	CES-D	Three good things	59	70	129	.10
Seligman.2005.1	prepost-msds	CES-D	You at your best	68	70	138	.10
Seligman.2005.1	prepost-msds	CES-D	Signature strengths	66	70	136	.07
Seligman.2005.1	prepost-msds	CES-D	Identifying signature strengths	68	70	138	.03
Seligman.2006.1	prepost-msds	BDI-II	Positive psychotherapy	14	20	34	.22
Seligman.2006.2	prepost-msds	ZSRS	Positive psychotherapy	11	9	20	.47
Seligman.2006.2	post-msds	HRSD	Positive psychotherapy	11	9	20	.59
Smith.1995.1*	prepost-difmsds	BDI	Personal happiness	17	12	29	.39
Smith.1995.1*	prepost-difmsds	BDI	Personal happiness w/ meditation	7	12	19	.60
Surawy.2005.1	prepost-msds	HADS-D	Mindfulness	9	8	17	.19
Zautra.2008.1a	prepost-msds	DEPS-NS	Mindfulness	41	30	71	-.03
Zautra.2008.1b	prepost-msds	DEPS-NS	Mindfulness	6	14	20	.31

Note

* = non-randomized study

*N*t = treatment sample size; *N*c = control sample size; *N*_total_ = total sample size; prepost-msds = pre and post means and standard deviations; BDI-II = Beck Depression Inventory-II; CES-D = Center for Epidemiologic Studies Depression Scale; SZD = Zung Scale for Depression; CID-DEP = Clinical Interview for Depression; SQ-DEP = Kellner's Symptom Questionnaire–Depression; post-msds = means and standard deviations from post data only; DAC = Depression Adjective Checklist; BDI = Beck Depression Inventory; HADS-D = Hospital Anxiety & Depression Scale–Depression; post-cohend–Cohen's d from post data only; ZSRS = Zung Self-Rating Scale for Depression; HRSD = Hamilton Rating Scale for Depression; prepost-difmsds = pre and post mean differences and standard deviations; DEPS-NS = Depressive Symptoms—not specified

In contrast to Sin and Lyubomirsky [22], Bolier et al. [23] addressed publication bias by computing the *Orwin’s fail-safe number*, and by using the *Trim and Fill method [32]*. Although the *Orwin’s fail-safe number* and *Trim and Fill* methods used to address publication bias are preferred over the *Fail-safe N* method, these approaches are limited and have been superseded by more advanced methods designed to estimate an effect size in the presence of small study bias including cumulative meta-analyses, the top 10%, and limit meta-analyses [33–35]. Thus, it is unclear whether a reanalysis of Sin and Lyubomirsky’s [22] and Bolier et al.’s [23] data, using more appropriate methods for taking into account small sample size effects, would confirm their findings or result in smaller effect size estimates.

Fifth, both Sin and Lyubomirsky and Bolier et al. also reported a number of group moderator analyses. Sin and Lyubomirsky reported six moderator analyses on well-being and six moderator analyses on depression. Similarly, Bolier et al. reported six moderator analyses on subjective well-being, six on psychological well-being, and six on depression. The inspection of these moderator analyses shows that groups consisted as few as two studies in Sin and Lyubomirsky (10 out of 12 moderator analyses included groups with 10 or fewer studies), and as few as one study in Bolier et al. (15 out of 16 moderator analyses included groups with 10 or fewer studies). Moreover, the number of studies in the moderator groups was widely discrepant for most of their moderator analyses. However, moderator analyses based on such a small number of studies in individual groups are not powerful enough to detect even large moderator effects [36]. Moreover, the power to detect moderator effects decreases still further when the number of studies in moderator groups is unequal [36]. Thus, in addition to the issues detailed above, the moderator analyses lacked the statistical power to make them meaningful.

Accordingly, the current study had two major objectives. The first objective was to reanalyze the reported data provided by the two meta-analyses while taking into account small sample size bias and comparing the findings to the original meta-analyses. The second objective was to replicate the two meta-analyses starting with extracting relevant data to calculate effect sizes directly from the primary studies rather than relying on the data published in the previous meta-analyses. In conducting these meta-analyses, the data were analyzed using weighted random effect models while taking into account small sample size bias using the selected methods discussed above.

## Method

### Primary studies

The primary studies selected for two major meta-analyses were included in the present study. Sin and Lyubomirsky [22] selected 49 primary studies on well-being [4,13,17,18,20,37–69] and 25 primary studies on depression [4,17,20,39,40,42–44,48,51,55,56,61,65,67,69–72]. Bolier et al. [23] selected 28 primary studies on subjective well-being [41,46,49,51,57,62,64,69,73–90], 20 primary studies on psychological well-being [4,17,20,41,42,46,57,62,64,76,80,82,83,90–99], and 14 primary studies on depression [4,17,20,42,51,62,77,78,82,91,93,96–100].

### Relevant data extraction and coding of primary studies

The selected primary studies used a variety of research designs (e.g., pre-post, post only), included one or more relevant interventions within the same study, and included one or more relevant outcome measures. Only interventions designed to improve well-being and/or decrease depression were considered relevant. Similarly, only measures of well-being and/or depression were relevant. Studies that included more than one intervention often employed only one control condition, which was used to determine the effectiveness of each intervention. Some studies included more than one control condition some of which were designed to decrease well-being and some were designed to increase well-being. Accordingly, we coded control conditions according to their presumed effect on well-being (negative, neutral, positive) and chose the most neutral control conditions to calculate PPI effect sizes. Thus, to calculate PPIs effect sizes, we extracted the following data for each study, intervention, and relevant outcome measure: research design (e.g., pre-post, post only); intervention; outcome measure; sample size of both control and intervention group; overall sample size; means and standard deviations of both pre and post assessments; within condition correlations between pre and post measurements (these were rarely provided); any *F*, *t*, *p*, or effect size (e.g., Cohen’s *d*) statistics reported for post only comparisons between control and intervention conditions; mean differences between pre and post measurements and associated standard deviations; and any other relevant data that allowed for effect size calculations.

### Effect size calculations

The primary studies that examined the effectiveness of interventions on well-being and/or depression symptoms used a variety of research designs, including repeated measures, pre-post designs, and between subjects post only measures designs. Although it is relatively straightforward to calculate effect sizes (i.e., *r*s or Cohen’s *d*s) for between subject post only designs using means, standard deviations, *F*s, *t*s, or *p*s, it is much more challenging to calculate effect sizes for repeated measures pre-post designs [26]. Primary studies using repeated measures pre-post designs rarely report sufficient statistical detail (such as the necessary correlations between pre and post scores), and thus, it is often necessary to impute estimated pre-post correlations using data obtained from other studies. Critically, it is not appropriate to use *F*s, *t*s, or *p*s to calculate effect sizes using formulae designed for between subject designs (i.e., formulae that do not take into account pre-post correlations). Accordingly, our initial approach was to calculate effect sizes for pre-post repeated measures designs using a formula recommended by Morris [101], specifically, *d*_ppc2_, using means, standard deviations, and when necessary, imputed pre-post correlations. Additionally, effect sizes were calculated using only post means and standard deviations, effectively treating these repeated measures pre-post designs as between subjects post-only designs. However, because the primary studies did not report pre-post correlations for outcome measures, it was not possible to calculate *d*_ppc2_ without imputing such correlations from elsewhere for each study.

Some primary studies used multiple outcome measures. To ensure that each study only contributed one effect size for each meta-analysis, effect sizes were first calculated for each outcome measure, and then aggregated to yield a single effect size. This was done while taking into account the correlations among the within-study outcomes using methods described by Schmidt and Hunter [102] and imputing a recommended default correlation of *r* = .50 for between within-study effects [103]. The aggregation of within-study outcomes was done using the R package MAc [104].

Similarly, some primary studies used multiple interventions. Moreover, only some of these interventions were designed within the positive psychology framework to improve well-being and/or decrease depression symptoms. Thus, effect sizes were calculated for each intervention designed to improve well-being and/or decrease depression symptoms within the positive psychology framework, and resulting effect sizes were aggregated to yield a single effect size from each study. For example, Emmons and McCullough [105] employed three experimental conditions: (a) participants listed things they were grateful for in their life, (b) participants listed hassles they encountered that day, and (c) participants listed events that happened during the week that impacted their life. In this case, the first condition (gratitude listing) was classified as the intervention group and the last condition (event listing) as the control group. As another example, Lyubomirsky, Dickerhoof, Boehm, and Sheldon [69] used three experimental conditions: (a) participants expressed optimism, (b) participants expressed gratitude, and (c) participants listed activities from the previous week. In this case, the first two conditions (optimism and gratitude) were classified as the intervention groups and the third condition was classified as the control group. Subsequently, the effect sizes obtained for the two interventions were aggregated into a single effect size for that particular study using methods recommended by Schmidt and Hunter [102] as described above.

Finally, some primary studies–seven in Sin and Lyubomirsky’s (2009) study set and three in Bolier et al.’s (2013) study set–used multiple control or comparison groups, ranging from interventions that may have decreased well-being (e.g., asking participants to reflect on negative experiences), to neutral controls, to interventions that increased well-being. In these cases, the most neutral control was chosen when calculating effect sizes. However, in some cases the control group was not clearly identified. For example, Low et al. [106] included three groups of female patients with breast cancer, who were asked to write about one of three possible options: (a) positive thoughts about their breast cancer experience, (b) deepest thoughts and feelings about their experience with breast cancer, and (c) facts about breast cancer and treatment. The first condition (positive thoughts) was classified as the intervention, which fits within the positive psychology framework, and the last condition (facts about breast cancer and its treatment) was used as the control. Finally, for studies by Cook [38] and Buchanan and Bardi [74], the no intervention controls were chosen over other controls, and for Tkach [107], the condition in which participants described any 3 events, 3 times a day, once a week was selected over other controls.

Effect sizes for primary study outcomes were calculated from available data in the following order of preference: (1) the post intervention means and standard deviations, (2) the post intervention ANOVA *F* values, (3) the post intervention Cohen's *d*s, (4) the post intervention *p* values, and (5) the pre-post difference score means and standard deviations as the difference between intervention and control effect sizes.

### Missing data and other irregularities

A number of primary studies included in the previous meta-analyses did not report sufficient data to calculate effect sizes. In the previous meta-analyses, the effects sizes for these studies were imputed to be zero (e.g., [46,57,68]). In the current replication analyses, such studies were excluded unless missing data could be imputed from other relevant sources. For example, if standard deviations for an outcome measure were missing in one study/experiment but were reported elsewhere (e.g., for another study/experiment within the same article), the missing standard deviations were imputed from the available ones to allow the calculation of effect sizes (e.g., Pretorious et al. [108]).

A number of primary studies only reported an overall sample size and did not report the sample size for the control and intervention groups. In such cases, the sample sizes for control and intervention groups were estimated by dividing the overall sample size by the number of control and intervention groups. Lastly, four articles–Shapira and Mongrain [96], Sergeant and Mongrain [99], Mongrain and Anselmo-Matthews [97], and Mongrain, Chin, and Shapira [109]–report on four seemingly different studies but actually report on different conditions/interventions of the same study. Accordingly, these four articles were treated as a single study.

### Statistical analyses

After all effect sizes were calculated, they were pooled to obtain a weighted effect size of PPIs using a random effects model. A random effects model was chosen because true PPI effects are unlikely to be the same and are likely to vary across the interventions, participants, and designs [33,110]. A fixed effect model meta-analysis assumes that all primary study effects estimate one common underlying true effect size. In contrast, a random effect model meta-analysis assumes that primary study effects may estimate different underlying true effect sizes (e.g., a true effect size may vary depending on participants’ age and the duration of the interventions).

Heterogeneity–variation or inconsistency found among effect sizes–is expected to be due to chance and to the array of interventions and samples used. Considerable heterogeneity indicates substantial differences between studies. To assess this, two common heterogeneity statistics were calculated: Cochran’s *Q [111]* and *I*^2^ [112]. The *Q* statistic employs a chi-square distribution *k* (number of studies)– 1 degrees of freedom—and only informs us of whether or not heterogeneity exists; it does not indicate how much heterogeneity exists and it is dependent on sample size. In contrast, the *I*^2^ statistic provides a percentage of total between-study variability found among the effects sizes, where a result of *I*^2^ = 0 means that the variability found among the estimated effects size is due solely to sampling error within studies [113].

Small study effects were assessed by first examining scatter plots, forest plots, and funnel plots. Several methods were used to estimate effect sizes while taking into account small study effects. First, the *Trim and Fill* procedure was used [32]. Second, a cumulative meta-analysis was used to determine how much the addition of small size studies would change the estimated effect size. Third, the effect sizes were estimated based on the top 10% (TOP10) of the most precise studies [114]. Stanley and Doucouliagos [114] demonstrated that the TOP10, despite its simplicity, performs well in estimating effect sizes in the presence of small sample size bias. Finally, the effect sizes were estimated using limit meta-analysis [115] which is the most sophisticated of the methods developed for estimating effect sizes in the presence of small sample size bias. The limit meta-analysis has been shown to be superior to other available methods including the trim-and-fill methods and selection models methods [116]. Accordingly, we report only the limit meta-analysis results. All analyses were conducted using R [117], including packages compute.es [118], MAc [104] meta [119], metafor [120], and metasens [121].

Following the procedure described in Cooper and Hedges [122], outliers were identified as effect sizes that were at least 1.5 times the interquartile range above the upper quartile or below the lower quartile of the distribution of effect sizes. When outliers were identified, a meta-analysis was re-run after removal of the outliers to assess the impact of outliers on the findings. Using the method for identifying outliers described by Viechtbauer and Cheung [123] yielded similar results.

### Moderator analyses

For the reasons detailed in the introduction, we have not attempted to reanalyze and replicate the moderator analyses published in Sin and Lyubomirsky (2009) and Bolier et al. (2013). Any such moderator analyses would be uninterpretable and not meaningful due to the small number of studies as well as the discrepant number of studies in the moderator groups [36]. Moreover, other issues reviewed in the introduction–most importantly the prevalent small sample size bias and non-comprehensive search for relevant primary studies–would also render any such analyses uninterpretable.

## Results

### Sin and Lyubomirsky (2009) meta-analysis

#### Well-being: Reanalysis of reported data

**Fig 1:**
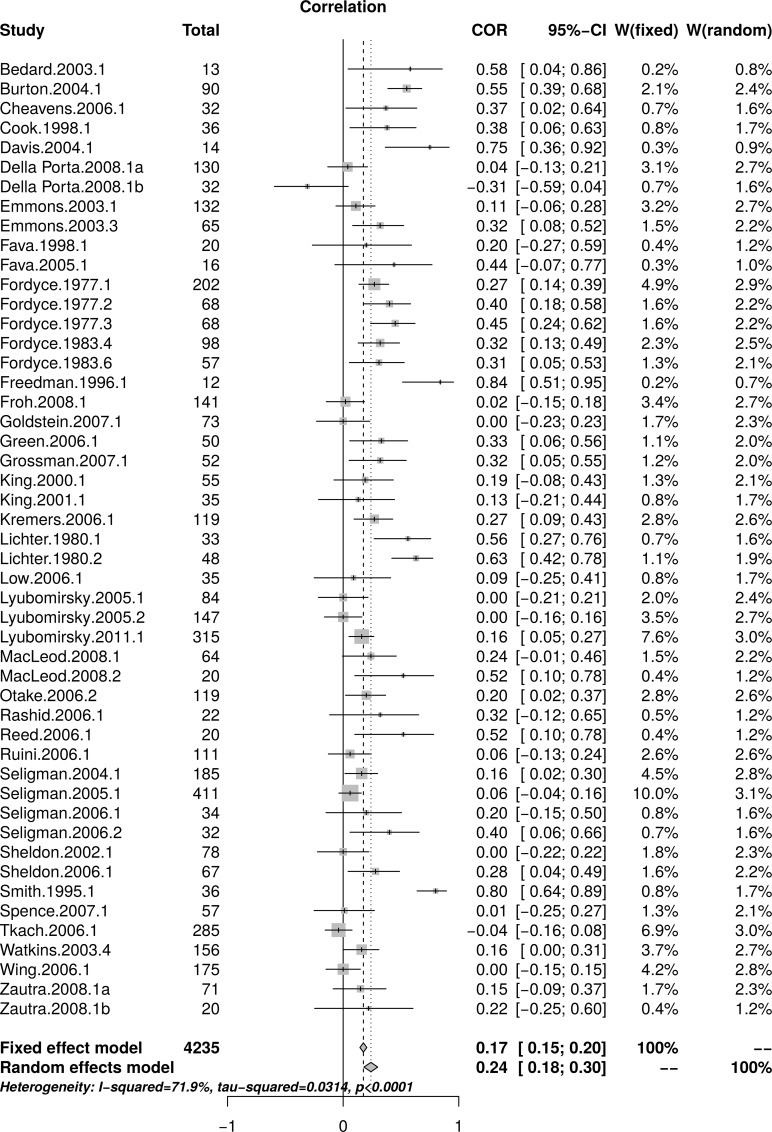
Reanalysis of Sin and Lyubomirsky (2009) well-being effect sizes: Forest plot of study effect sizes. The forest plot indicates substantial scatter among the effect sizes and suggests that small studies resulted in larger effect sizes than large studies.

The reanalysis used data reported by Sin and Lyubomirsky [22] in their Table 1. Fig 1 shows the forest plot of effect sizes (*r*s) as reported by Sin and Lyubomirsky, including total sample size for each study in the “Total” column. The forest plot indicates that small studies resulted in larger effect sizes than large studies. A random effect model estimated an effect size of *r* = .24 [95% CI = (0.18, 0.30)] with substantial heterogeneity as measured by *I*^2^ = 71.9%.

**Fig 2:**
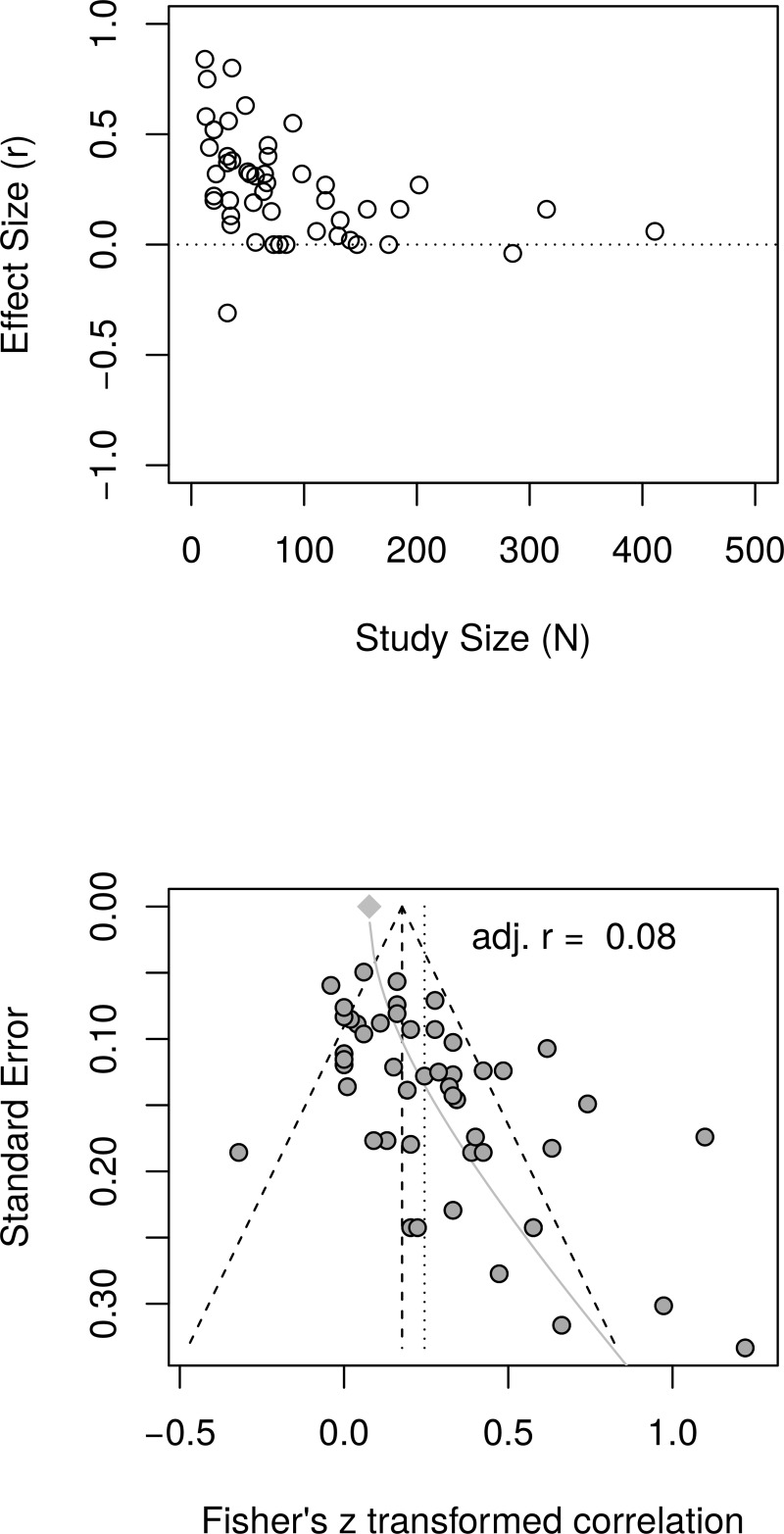
Reanalysis of Sin and Lyubomirsky (2009) well-being effect sizes: Relationship between study sizes and effect sizes. The top panel shows the scatter plot of effect sizes by study sizes. The bottom panel shows the funnel plot and the results of the limit meta-analysis including the estimated effect size taking into account small-study effect.

Fig 2, top panel, shows a scatter plot of effect sizes and study sizes. The scatter plot indicates the presence of a small study effect. Fig 2, bottom panel, shows the funnel plot with substantial asymmetry. The regression test of the funnel plot symmetry confirmed that the plot was asymmetrical, *t*(47) = 4.46, *p* < .001. Accordingly, we estimated the effect size after accounting for the small study size bias. The limit meta-analyses (Fig 2, bottom panel) resulted in an effect size of *r* = .08 [95% CI = (0.00, 0.15)]. A test of small-study effects showed *Q-Q'*(1) = 50.83, *p* < .001. A test of residual heterogeneity indicated *Q*(47) = 120.24, *p* < .001. Thus, taking into account small study effects, the reanalyses resulted in a much smaller estimated effect size for well-being than the effect size (*r* = .29) reported by Sin and Lyubomirsky [22].

#### Well-being: Complete replication of meta-analysis

Table 1 reports effect sizes for PPIs on well-being determined as described above for each outcome measure and each intervention comparison. These effect sizes were then aggregated to yield a single effect size for each study comparable to those reported in Sin and Lyubomirsky [22] using the aggregation method described in the Method section. The correlation between the effect sizes reported by Sin and Lyubomirsky [22] and the effect sizes calculated through this replication was high, *r* = .78 [95% CI = (0.62, 0.88)].

**Fig 3:**
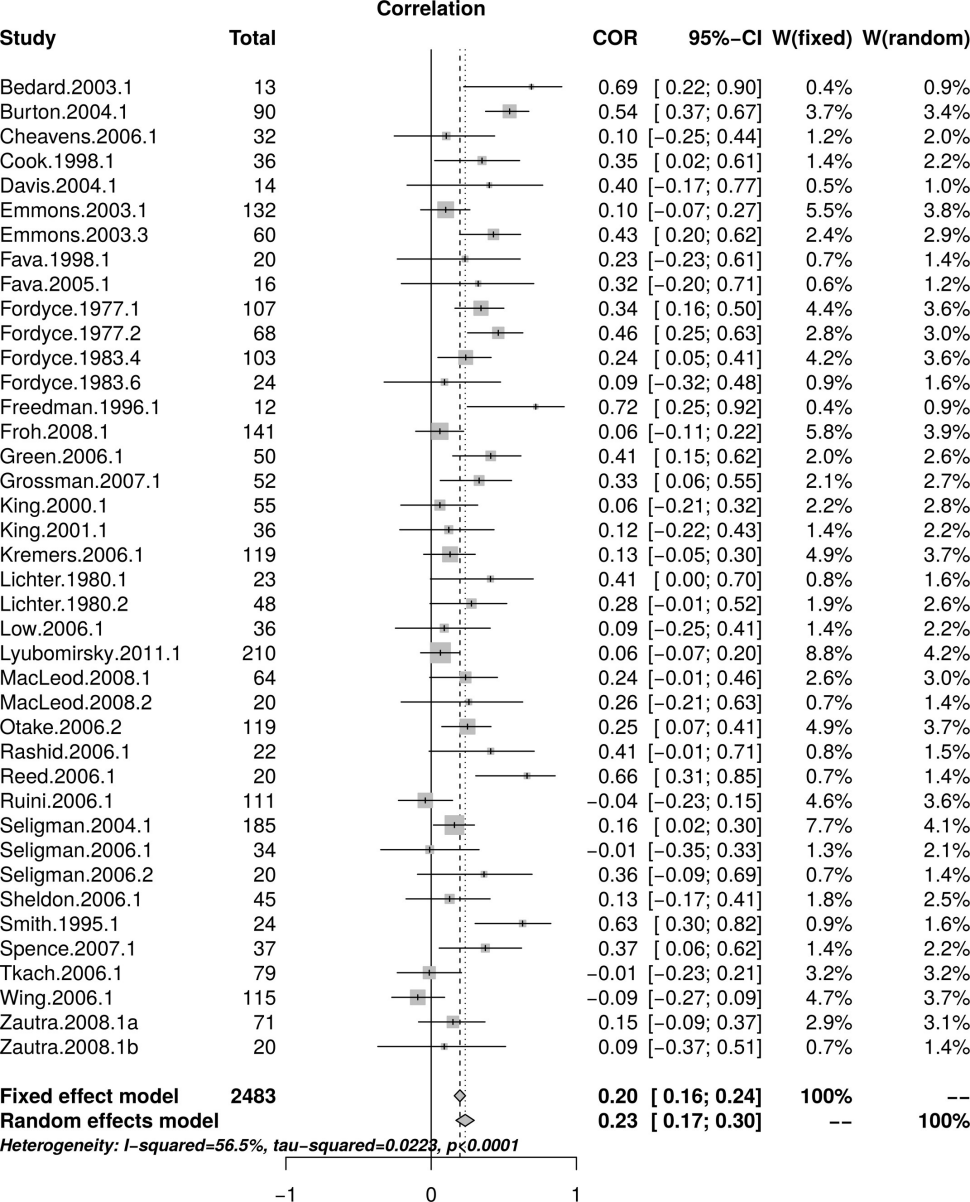
Complete replication of Sin and Lyubomirsky (2009) well-being effect sizes: Forest plot of study effect sizes. The forest plot indicates substantial scatter among the effect sizes and suggests that small studies resulted in larger effect sizes compared to larger studies.

**Fig 4:**
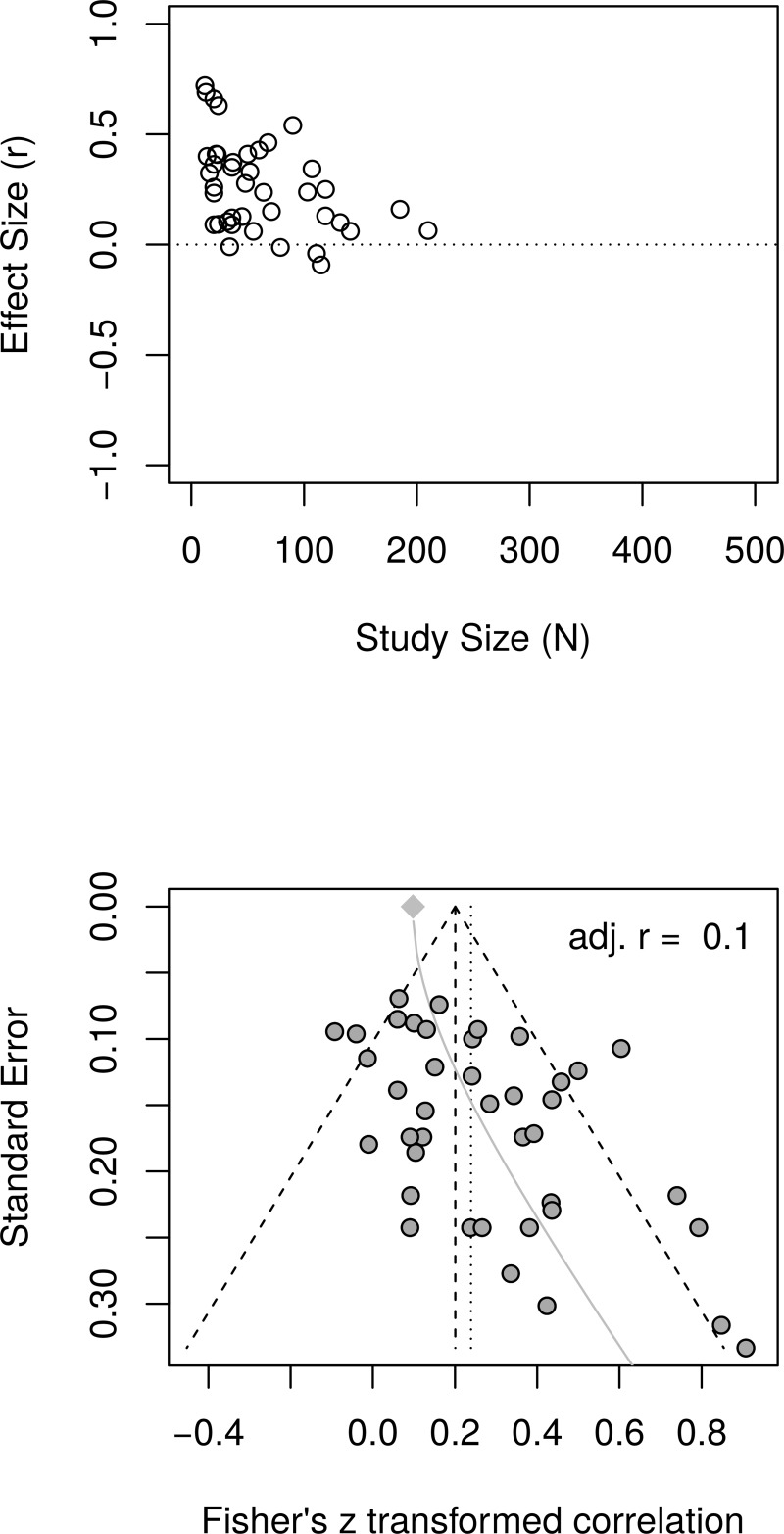
Complete replication of Sin and Lyubomirsky (2009) well-being effect sizes: Relationship between study sizes and effect sizes. The top panel shows the scatter plot of effect sizes by study sizes. The bottom panel shows the funnel plot and the results of the limit meta-analysis including the estimated effect size taking into account small-study effect.

Fig 3 shows the forest plot of the replication effect sizes and suggests that small studies reported larger effects than large studies. A random effect model estimated an effect size of *r* = .23 [95% CI = (0.17, 0.30)] with moderate heterogeneity as measured by *I*^2^ = 56.5%. Fig 4, top panel, shows a scatter plot of effect sizes and study sizes. The scatter plot indicates the presence of a small study effect. Fig 4, the bottom panel, shows the funnel plot with substantial asymmetry. The regression test of the funnel plot symmetry confirmed that the plot was asymmetrical, *t*(38) = 3.19, *p* = .003. Accordingly, we estimated the effect size after accounting for the small study size bias. The limit meta-analyses (bottom of Fig 4) estimated an effect size of *r* = .10 [95% CI = (-0.01, 0.20)]. A test of small-study effects showed *Q-Q'*(1) = 18.89, *p* < .001 and a test of residual heterogeneity indicated that *Q*(38) = 70.68, *p* < .001. Thus, similar to the reanalyses of Sin and Lyubomirsky’s [22] data, the replication resulted in a much smaller effect size estimate than that originally reported by Sin and Lyubomirsky (*r* = .29).

#### Depression: Reanalysis of reported data

**Fig 5:**
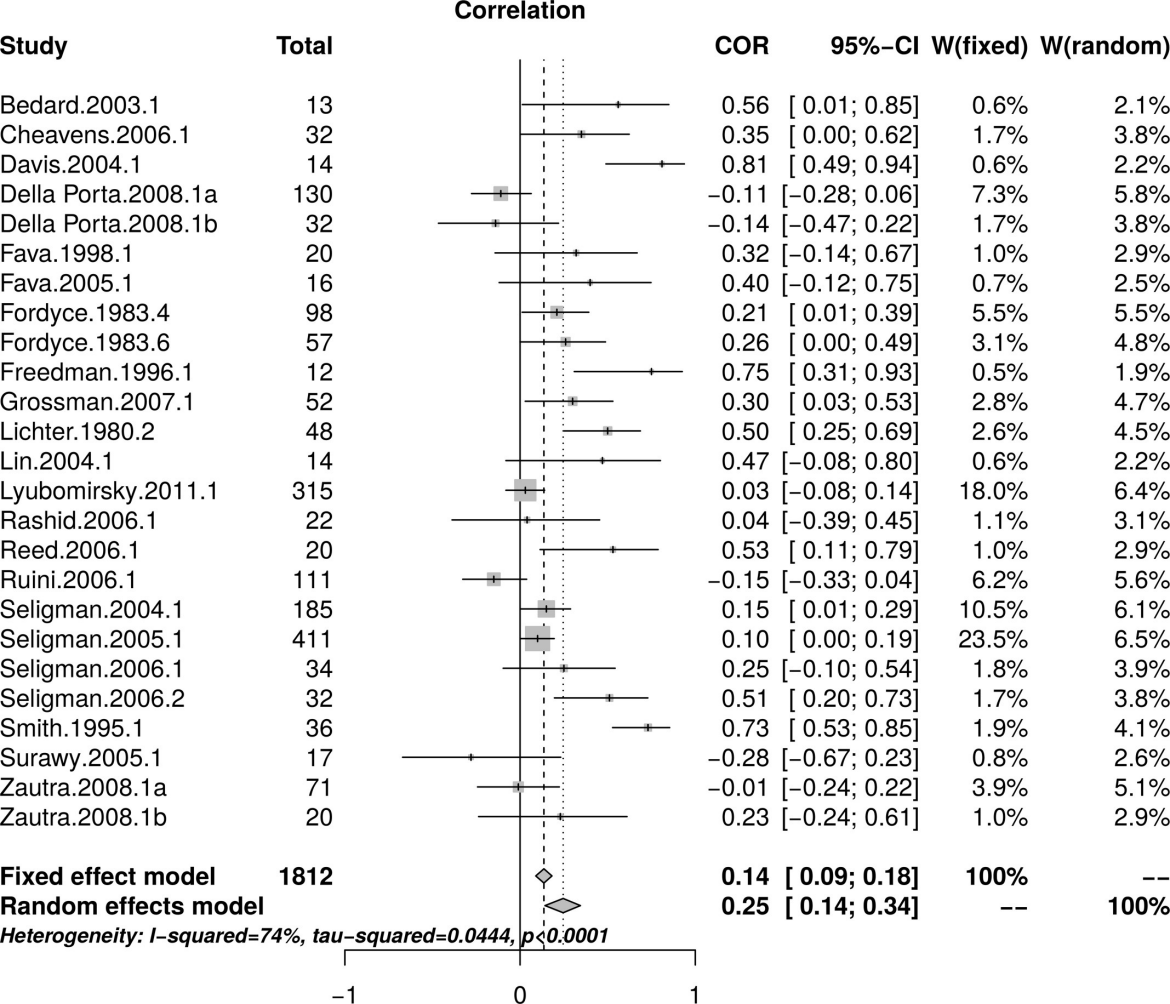
Reanalysis of Sin and Lyubomirsky (2009) depression effect sizes: Forest plot of study effect sizes. The forest plot indicates substantial scatter among the effect sizes and suggests that small studies resulted in larger effects than large studies.

The reanalysis used data reported by Sin and Lyubomirsky [22] in their Table 2. Fig 5 shows the forest plot of effect sizes. Again, the forest plot indicates that small studies reported larger effects than large studies. A random effect model estimated an effect size of *r* = .25 [95% CI = (0.14, 0.34)] with substantial heterogeneity as measured by *I*^2^ = 74%.

**Fig 6:**
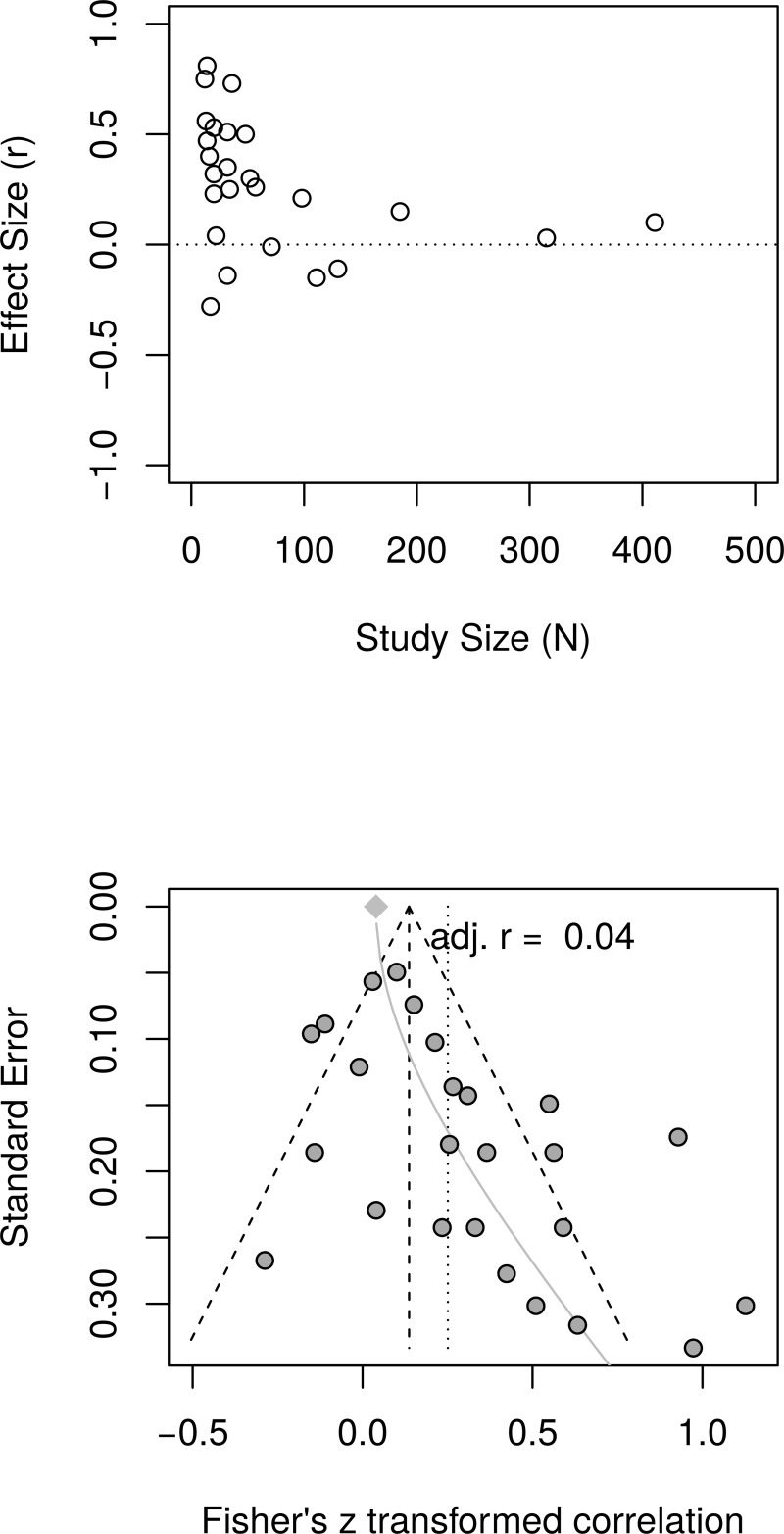
Reanalysis of Sin and Lyubomirsky (2009) depression effect sizes: Relationship between study sizes and effect sizes. Top panel shows the scatter plot of effect sizes by study sizes. The bottom panel shows the funnel plot and the results of the limit meta-analysis including the estimated effect size taking into account small study effects.

Fig 6, top panel, shows the scatter plot of effect sizes and study sizes. The scatter plot indicates the presence of small study effects. Fig 6, bottom panel, shows the funnel plot with substantial asymmetry. The regression test of the funnel plot symmetry confirmed that the plot was asymmetrical, *t*(23) = 3.20, *p* = .004. Accordingly, we estimated the effect size after accounting for the small study size bias. The limit meta-analysis (Fig 6, bottom panel) resulted in effect size of *r* = .04 [95% CI = (-0.05, 0.13)]. A test of small-study effects showed *Q-Q'*(1) = 28.40, *p* < .001 and a test of residual heterogeneity indicated *Q*(23) = 63.79, *p* < .001. Thus, similar to the reanalysis of well-being effect sizes, taking into account small study effects, the reanalysis of depression effect sizes resulted in a much smaller, and now non-significant estimated effect size of PPIs on depression compared to the effect size (*r* = .31) reported by Sin and Lyubomirsky.

#### Depression: Complete replication of meta-analysis

Table 2 reports effect sizes for studies that assessed depression. The effect sizes were determined as described above for each outcome measure and each intervention comparison. These effect sizes were then aggregated to yield a single effect size for each study comparable to those reported in Sin and Lyubomirsky [22] using the aggregation method described in the Method section. The correlation between the effect sizes reported by Sin and Lyubomirsky [22] and the effect sizes calculated through this replication was high, *r* = .78 [95% CI = (0.52, 0.91).

**Fig 7:**
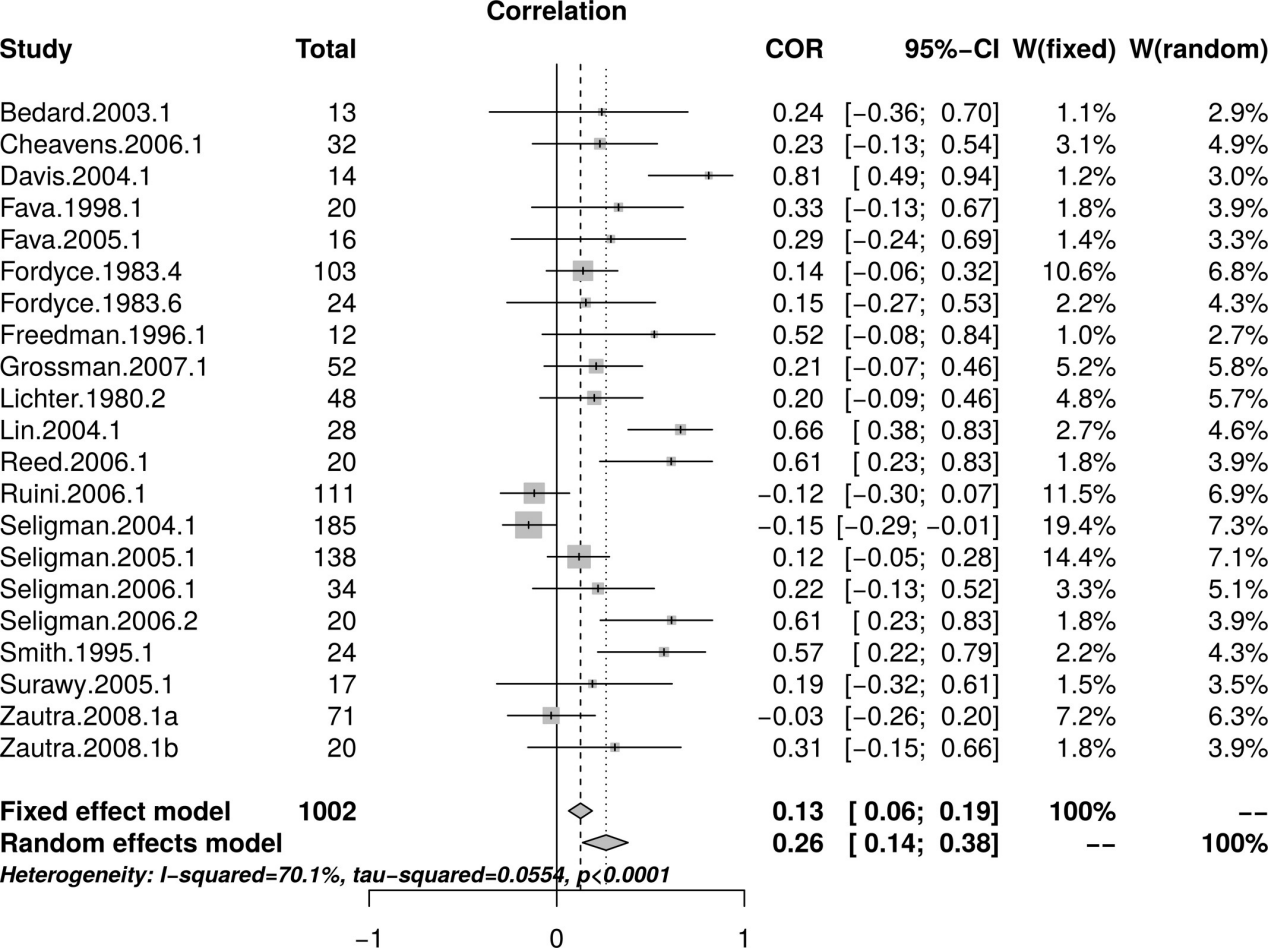
Complete replication of Sin and Lyubomirsky (2009) depression effect sizes: Forest plot of study effect sizes. The forest plot indicates substantial scatter among the effect sizes and suggests that small studies resulted in larger effect sizes than large studies.

**Fig 8:**
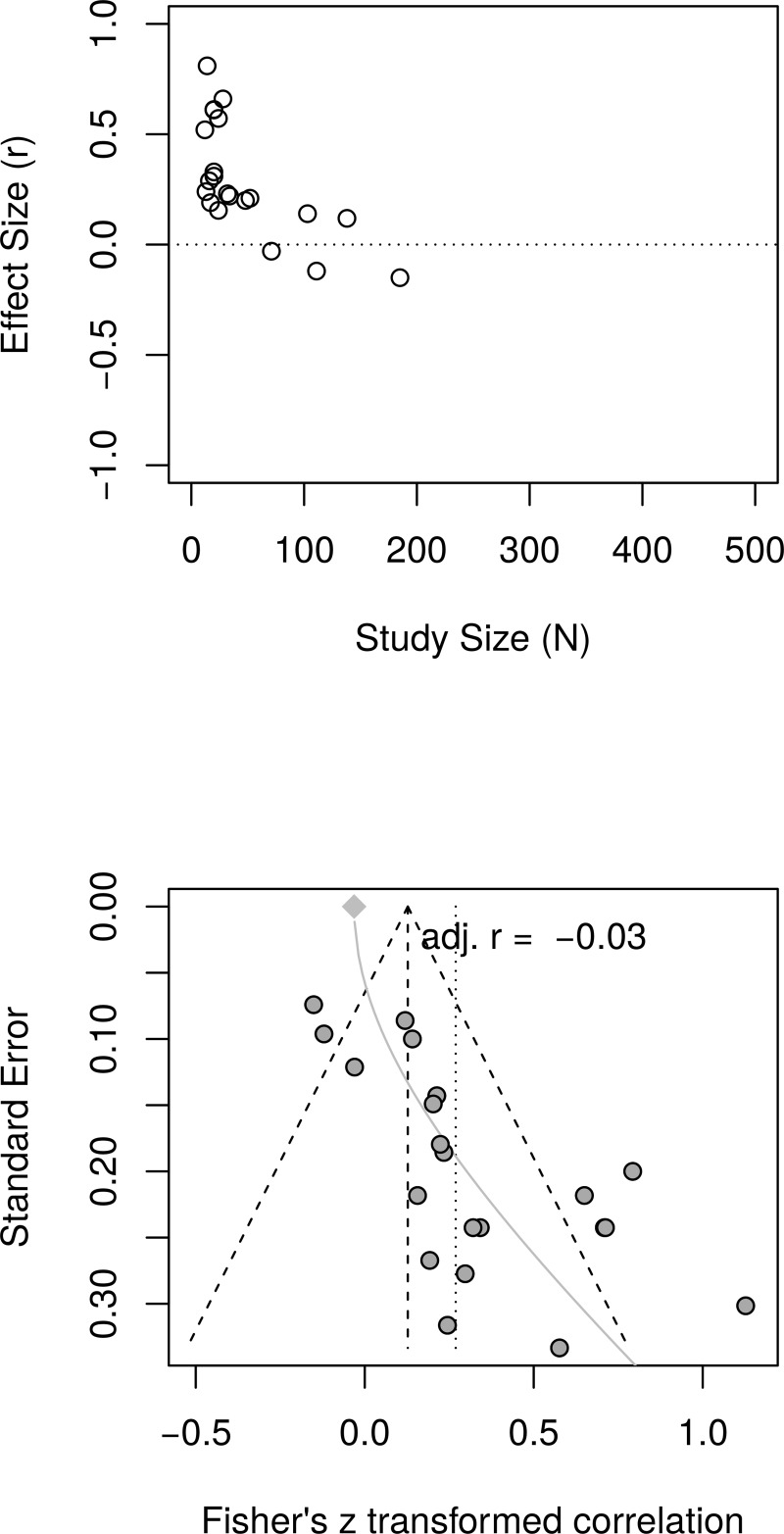
Complete replication of Sin and Lyubomirsky (2009) depression effect sizes: Relationship between study sizes and effect sizes. The top panel shows the scatter plot of effect sizes by study sizes. The bottom panel shows the funnel plot and the results of the limit meta-analysis including the estimated effect size taking into account small study effects.

Fig 7 shows the forest plot of the replication effect sizes. Again, the forest plot indicates that small studies resulted in larger effects than large studies. A random effect model estimated an effect size of *r* = .26 [95% CI = (0.14, 0.38)] with substantial heterogeneity as measured by *I*^2^ = 70.1%. Fig 8, top panel, shows a scatter plot of effect sizes by study sizes. The scatter plot indicates the presence of small study effects. Fig 8, bottom panel, shows the funnel plot with substantial asymmetry. The regression test of the funnel plot symmetry confirmed that the plot was asymmetrical, *t*(19) = 5.33, *p* < .001. Accordingly, we estimated the effect size in the presence of the small study size bias. The limit meta-analyses (Fig 8, bottom panel) estimated an effect size of *r* = -.03 [95% CI = (-0.17, 0.11)]. A test of small-study effects showed *Q-Q*'(1) = 40.06, *p* < .001 and a test of residual heterogeneity showed *Q*(19) = 26.82, *p* = .109. Thus, similar to the re-analysis of depression effect sizes, taking into account small study effects, the replication analyses resulted in a much smaller, and now non-significant estimated effect of PPIs on depression compared to the effect size reported by Sin and Lyubomirsky (*r* = .31).

### Bolier et al. (2013) meta-analysis

#### Subjective well-being: Reanalysis of reported data

**Fig 9:**
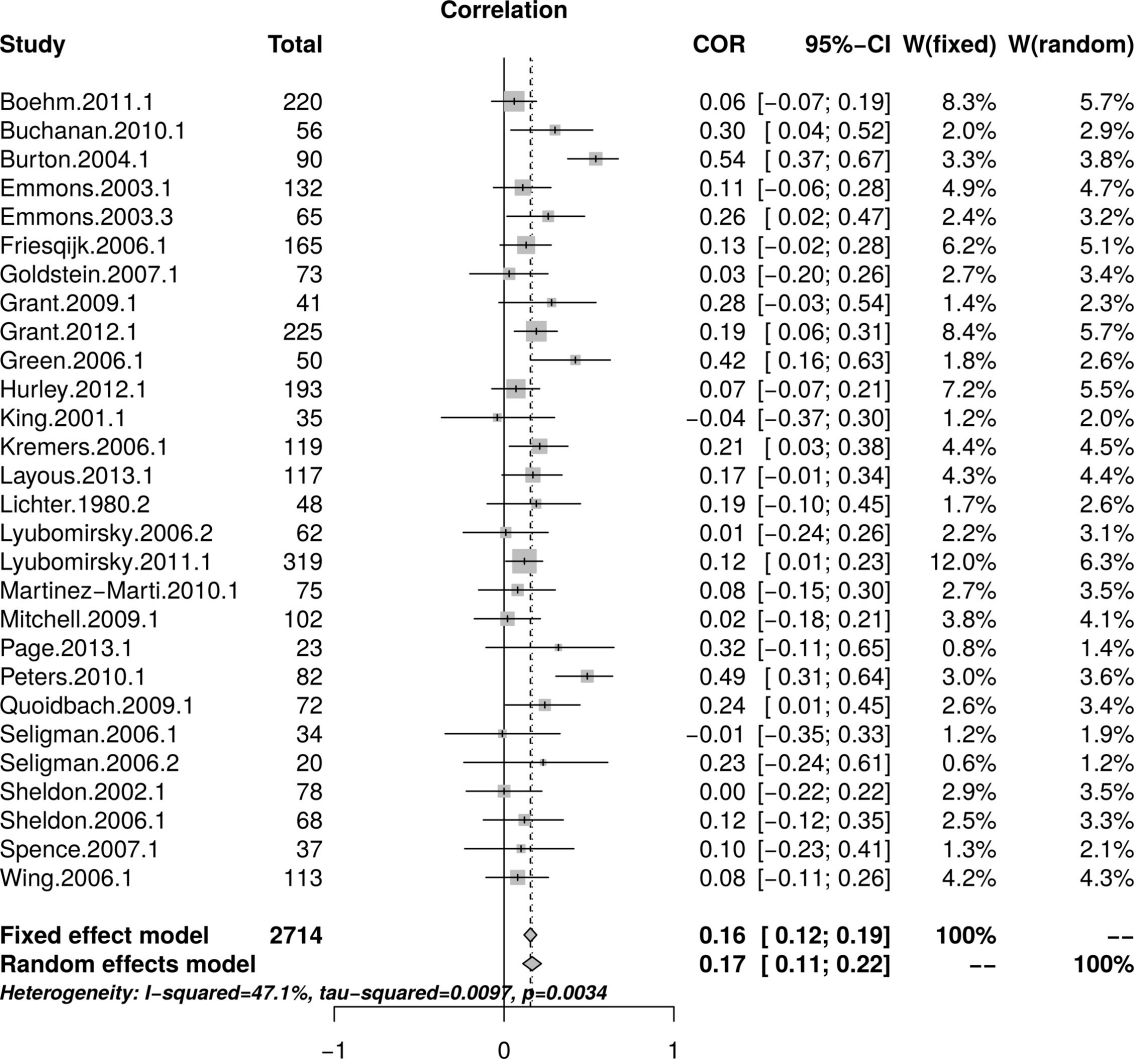
Reanalysis of Bolier et al. (2013) subjective well-being effect sizes: Forest plot of study effect sizes. The forest plot indicates some scatter among the effect sizes but suggests no consistent relationship between effect sizes and study sizes.

The reanalysis used data reported by Bolier et al. [23] in their Table 2 and Fig 2. Fig 9 shows the forest plot of effect sizes reported by Bolier et al. [23]. The forest plot reveals no obvious relationship between effect sizes and study sample sizes. The random effects model estimated an effect size of *r* = .17 [95% CI = (0.11, 0.22)] with moderate heterogeneity as measured by *I*^2^ = 47.1%.

**Fig 10:**
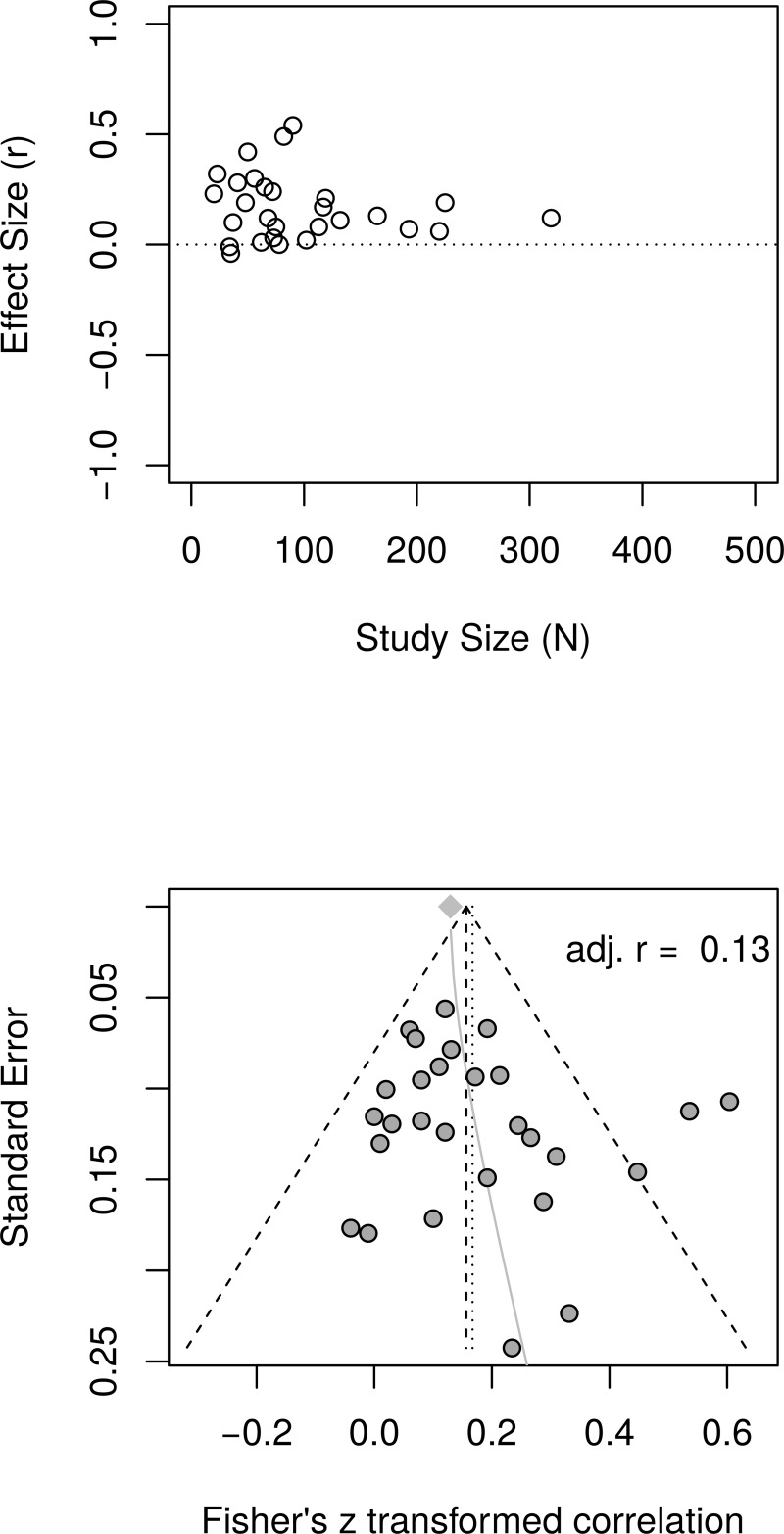
Reanalysis of Bolier et al. (2013) well-being effect sizes: Relationship between study sizes and effect sizes. The top panel shows the scatter plot of effect sizes by study sizes. The bottom panel shows the funnel plot and the results of the limit meta-analysis taking into account any small study effects.

Fig 10, top panel, shows the scatter plot of effect sizes as a function of study size and indicates no obvious relationship between effect sizes and study sizes. Fig 10, bottom panel, shows the funnel plots, which do not illustrate asymmetry. The regression test of the funnel plot symmetry was not statistically significant, *t*(26) = 1.06, *p* = .299. Furthermore, the limit meta-analyses (Fig 10, bottom panel) estimated an effect size of *r* = .13 [95% CI = (0.02, 0.24)], comparable to a random effect model without any adjustments. A test of small study effects showed *Q-Q*'(1) = 2.12, *p* = .145 and a test of residual heterogeneity indicated *Q*(26) = 48.96, *p* = .004. The reanalysis of Bolier et al.’s [23] subjective well-being data confirmed their findings.

#### Subjective well-being: Complete replication of meta-analysis

Table 3 reports effect sizes determined as described above for each outcome measure and intervention comparison. These effect sizes were aggregated to yield a single effect size for each study comparable to those reported in Bolier et al. [23]. The correlation between the effect sizes reported by Bolier et al. [23] and the effect sizes calculated through this replication was high, *r* = .85 [95% CI = (0.68, 0.94).

**Table 3:** Effect sizes determined by the current study, for each subjective well-being measure and each study included in Bolier et al. (2013) subjective well-being meta-analysis.

Study	Available Data	Measure	PPI condition	*N*_t_	*N*_c_	*N*_total_	*r*
Buchanan.2010.1	post-msds	SWLS	Acts of kindness	28	28	56	.34
Burton.2004.1	post-msds	PA-NS	Positive experiences	48	42	90	.54
Emmons.2003.1	post-msds	PA-NS	Gratitude	65	67	132	.10
Emmons.2003.3	post-anovaF	PA-NS	Gratitude	33	32	65	.27
Emmons.2003.3	post-anovaF	global life appraisals	Gratitude	33	32	65	.42
Emmons.2003.3	post-anovaF	expectations—upcoming week	Gratitude	33	32	65	.28
Emmons.2003.3	post-tpvalue	PANAS-P-observer	Gratitude	26	26	52	.26
Friesqijk.2006.1	prepost-msds	SPF-IL	Self-management	79	86	165	.13
Grant.2009.1	prepost-msds	WWBI	Leadership development	20	21	41	.16
Grant.2012.1	prepost-msds	PANAS-P	Solution coaching	117	108	225	.19
Green.2006.1	prepost-msds	SWLS	Solution coaching	23	25	48	.45
Green.2006.1	prepost-msds	PANAS-P	Solution coaching	25	25	50	.39
Hurley.2012.1	prepost-msds	PANAS-X-P	Savoring the moment	94	99	193	.07
King.2001.1	post-msds	D&E-NP	Best possible self	19	16	35	-.04
King.2001.1	post-msds	D&E-NP	Write about trauma + best possible self	22	16	38	.25
Kremers.2006.1	prepost-msds	SPFILS	Self-management	46	73	119	.13
Layous.2013.1	prepost-difmsds	AAS-P	Best possible self	80	37	117	.13
Lichter.1980.2	prepost-msds	HAP-AFFECT	Positive feeling statements	25	23	48	.19
Lyubomirsky.2006.2	post-msds	SWLS	Write about best experience	24	36	60	-.14
Lyubomirsky.2006.2	post-msds	PANAS-P	Write about best experience	24	36	60	-.04
Lyubomirsky.2006.2	post-msds	SWLS	Talk about best experience	25	36	61	-.32
Lyubomirsky.2006.2	post-msds	PANAS-P	Talk about best experience	25	36	61	-.03
Lyubomirsky.2006.2	post-msds	SWLS	Think about best experience	26	36	62	.12
Lyubomirsky.2006.2	post-msds	PANAS-P	Think about best experience	26	36	62	-.10
Lyubomirsky.2011.1	prepost-difmsds	UPL+PL+SWLS+SHS	Gratitude	107	101	208	.08
Lyubomirsky.2011.1	prepost-difmsds	UPL+PL+SWLS+SHS	Optimism	111	101	212	.03
Martinez-Marti.2010.1	prepost-msds	PA-NS	Gratitude	41	34	75	.15
Mitchell.2009.1	prepost-msds	PWI-A	Signature strengths	17	23	40	.09
Mitchell.2009.1	prepost-msds	SWLS	Signature strengths	17	23	40	-.06
Mitchell.2009.1	prepost-msds	PANAS-P	Signature strengths	17	23	40	.05
Page.2013.1	prepost-msds	SWLS + PANAS-P–PANAS-N	Wellness	23	14	37	.16
Page.2013.1	prepost-msds	AWB	Wellness	23	14	37	.57
Peters.2010.1	prepost-msds	PANAS-Short-P	Positive future thinking	44	38	82	.49
Seligman.2006.1	prepost-msds	SWLS	Positive psychotherapy	14	20	34	-.01
Seligman.2006.2	prepost-msds	SWLS	Positive psychotherapy	11	9	20	.23
Shapira.2010.1	prepost-msds	SHI	Self-compassion	63	70	133	.01
Shapira.2010.1	prepost-msds	SHI	Optimism	55	70	125	.11
Sheldon.2006.1	prepost-msds	PANAS-P	Gratitude	21	23	44	-.08
Sheldon.2006.1	prepost-msds	PANAS-P	Best possible self	23	23	46	.30
Spence.2007.1	prepost-msds	SWLS	Professional coaching	20	17	37	.38
Spence.2007.1	prepost-msds	B-PA	Professional coaching	20	17	37	.16
Spence.2007.1	prepost-msds	SWLS	Peer coaching	20	17	37	.38
Spence.2007.1	prepost-msds	B-PA	Peer coaching	20	17	37	.25
Wing.2006.1	prepost-msds	SWLS	Positive experiences w/cue	58	55	113	-.11
Wing.2006.1	prepost-msds	SWLS	Positive experiences	62	55	117	-.05

*Note*. *N*t = treatment sample size; *N*c = control sample size; *N*_total_ = total sample size; post-msds = means and standard deviations from post data only; SWLS = Satisfaction with Life Scale; PA-NS = Positive Affect, not specified; post-anovaF = anova F statistic from post data only; post-tpvalue = t statistic and p value from post data only; PANAS-P-observer = Positive and Negative Affect Schedule—Positive–Observer; prepost-msds = pre and post means and standard deviations; SPF-IL = Subjective Well-being; WWBI = Workplace Well-being Index; PANAS-P = Positive and Negative Affect Schedule–Positive; PANAS-X-P = Positive and Negative Affect Schedule—Expanded—Positive Affect; D&E-NP = Diener & Emmons Net Positive Mood; SPFILS = Social Production Function Index Level Scale; prepost-difmsds = pre and post mean differences and standard deviations; AAS-P = Affect-Adjective Scale—positive affect; HAP-AFFECT = Happiness—Affectometer 1; UPL+PL+SWLS+SHS = unpleasant affect, pleasant affect, SWLS, and SHS combined; PWI-A = Personal Well-Being Index; AWB = The Affective Well-Being; PANAS-Short-P = Positive and Negative Affect Schedule—Short—Positive Affect; SHI = Steen Happiness Index; B-PA = Bradburn—Positive Affect

**Fig 11:**
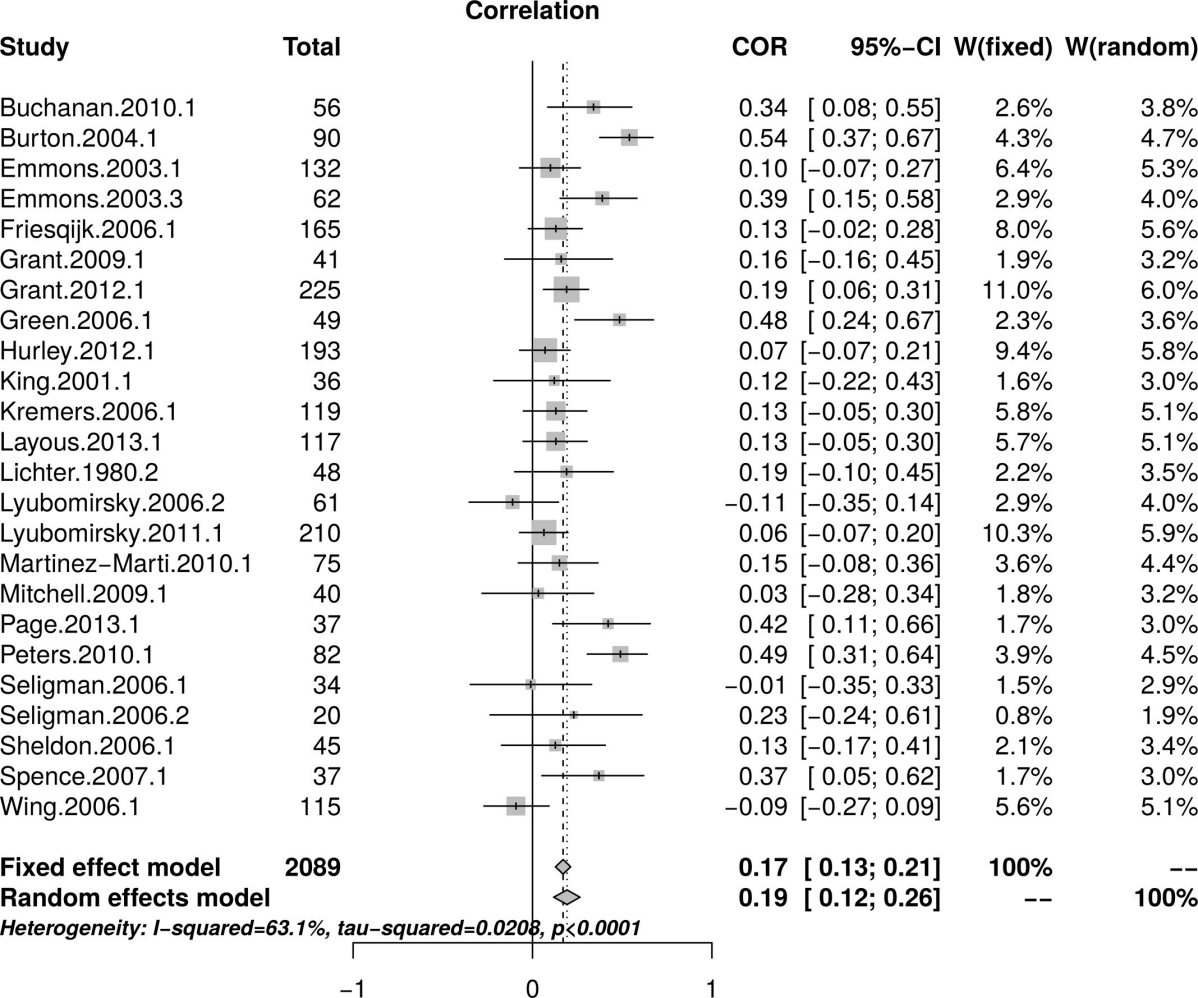
Complete replication of Bolier et al. (2013) subjective well-being effect sizes: Forest plot of study effect sizes. The forest plot indicates some scatter among the effect sizes but suggests no obvious relationship between effect sizes and study sizes.

**Fig 12:**
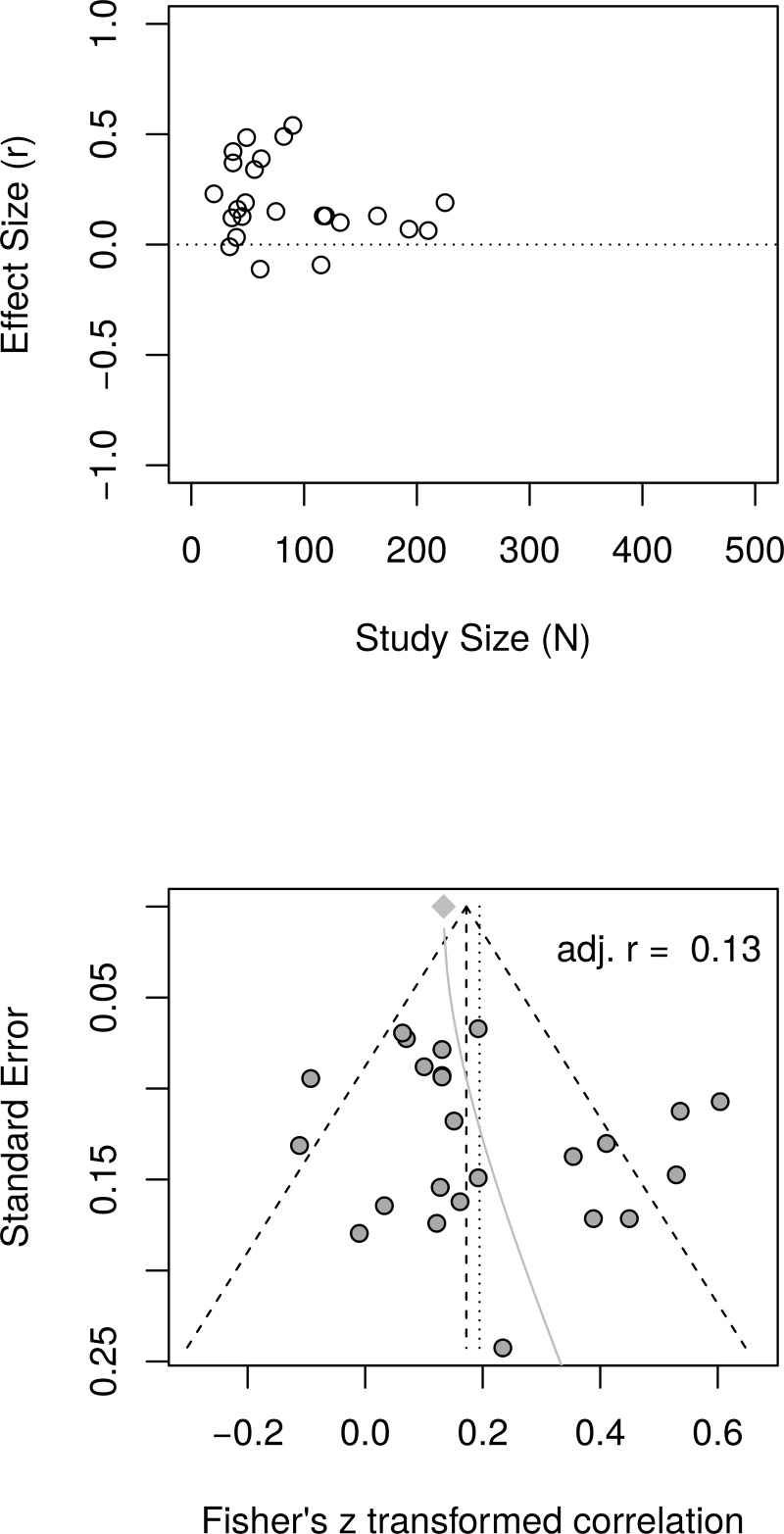
Complete replication of Bolier et al. (2013) subjective well-being effect sizes: Relationship between study sizes and effect sizes. The top panel shows the scatter plot of effect sizes by study sizes. The bottom panel shows the funnel plot and the results of the limit meta-analysis taking into account any small study effects.

Fig 11 shows the forest plot of effect sizes with no obvious signs of small study effects. A random effects model estimated an effect size of *r* = .19 [95% CI = (0.12, 0.26)] with moderate heterogeneity as measured by *I*^2^ (63.1%). Fig 12, top panel, shows the scatter plot of effect sizes by study size and indicates no obvious relationship between them. Fig 12, bottom panel, shows the funnel plot with no obvious asymmetry. The regression test of funnel plot symmetry was not statistically significant, *t*(22) = 1.37, *p* = .184. Furthermore, the limit meta-analyses (Fig 12, bottom panel) estimated an effect size of *r* = .13 [95% CI = (0.00, 0.26)]. A test of small-study effects showed *Q-Q'*(1) = 4.91, *p* = .027 and a test of residual heterogeneity indicated *Q*(22) = 57.39, *p* < .001. These results are similar to those reported by Bolier et al. [23] and obtained by the reanalysis of Bolier et al.’s data.

#### Psychological well-being: Reanalysis of reported data

**Fig 13:**
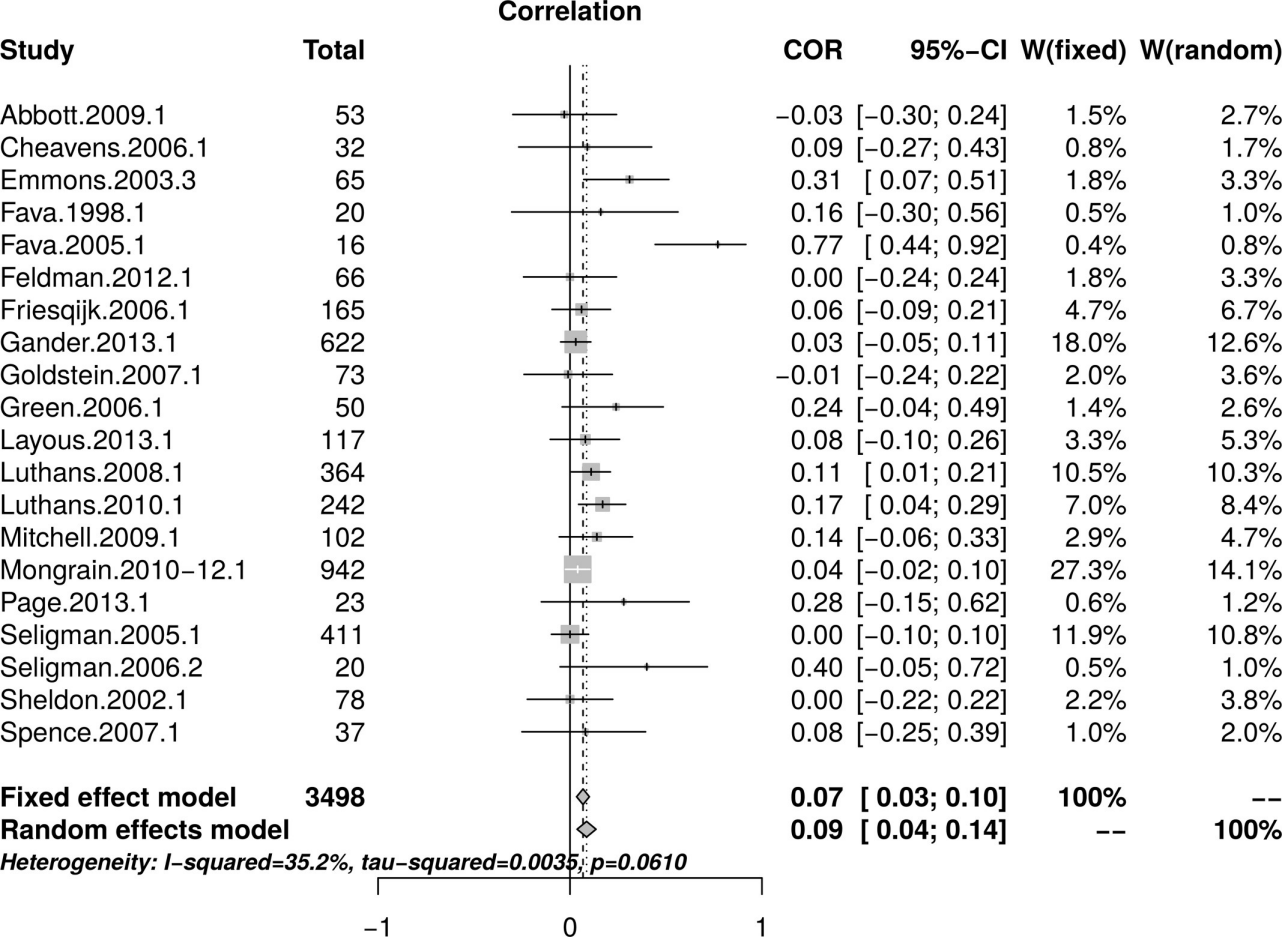
Reanalysis of Bolier et al. (2013) psychological well-being effect sizes: Forest plot of study effect sizes. The forest plot indicates that smaller studies reported large effect sizes than larger studies and also indicates the presence of a possible outlier (Fava.2005.1).

The reanalysis used data reported by Bolier et al. [23] in their Table 2 and Fig 3. Fig 13 shows the forest plot of effect sizes. The plot indicates the presence of small study effect and the presence of an outlier (Fava.2005.1). A random effect model estimated an effect size of *r* = .09 [95% CI = (0.04, 0.14)] with heterogeneity, as measured by *I*^2^, = 35.2%.

**Fig 14:**
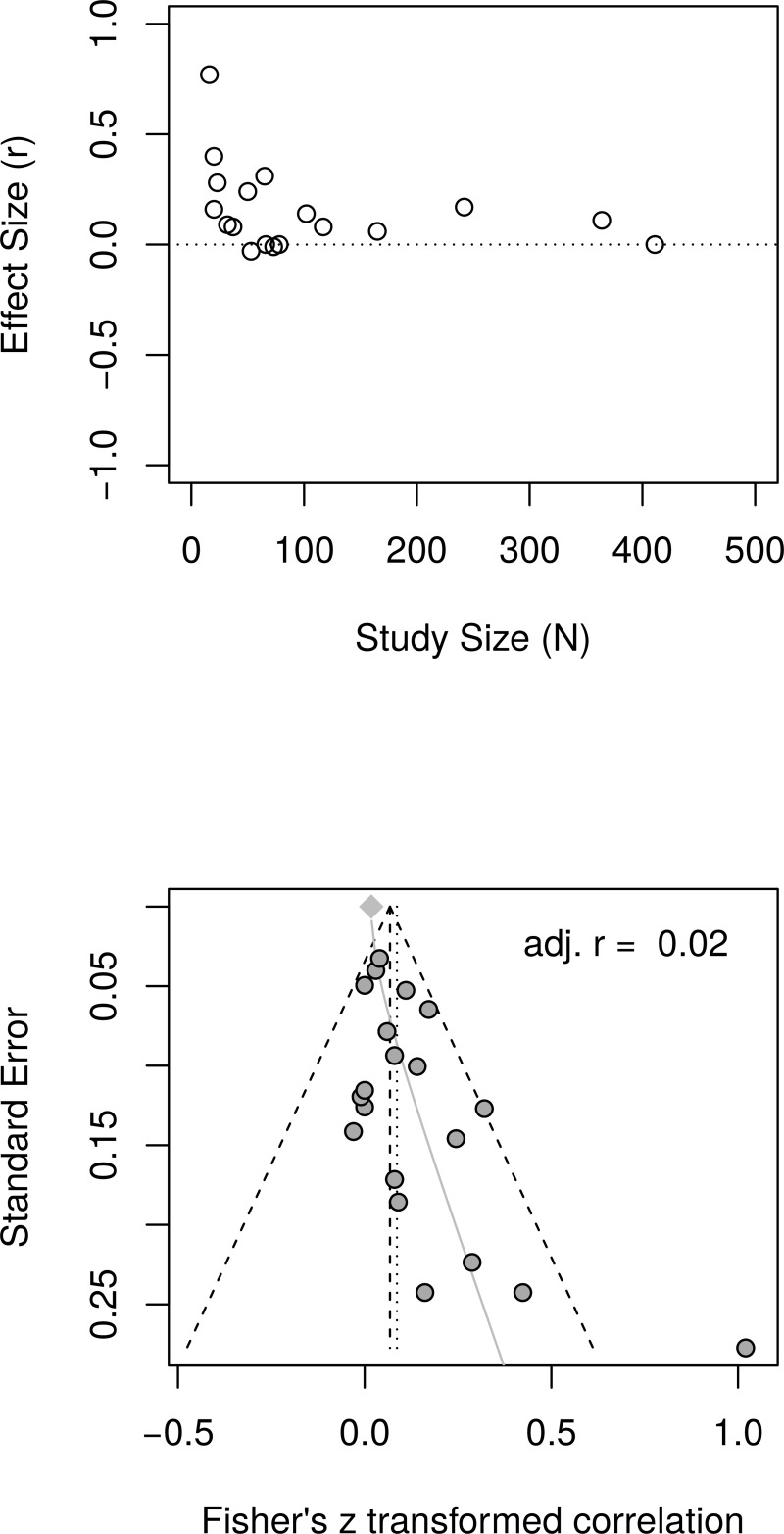
Reanalysis of Bolier et al. (2013) psychological well-being effect sizes: Relationship between effect sizes and study sizes. The top panel shows the scatter plot of effect sizes by study sizes. The bottom panel shows the funnel plot and the results of the limit meta-analysis taking into account any small study effects.

Fig 14, top panel, shows the scatter plot of effect sizes by study size and indicates the presence of small study effects. Fig 14, bottom panel, shows the funnel plot with visible asymmetry. The regression test of the funnel plot symmetry confirmed that the plot was asymmetrical, *t*(18) = 2.68, *p* = .02. Accordingly, it is necessary to estimate the effect size in the presence of the small study size bias. The limit meta-analyses (Fig 14, bottom panel) estimated an effect size of *r* = .02 [95% CI = (-0.04, 0.08)]. A test of small-study effects showed *Q-Q*'(1) = 8.36, *p* = .004 and a test of residual heterogeneity indicated *Q*(18) = 20.97, *p* = .281.

The analysis was recalculated after removing one outlier (Fava.2005.1). A random effect model estimated an effect size of *r* = .06 [95% CI = (0.03, 0.10)] with no heterogeneity, as measured by *I*^2^, = 0%. The regression test of the funnel plot symmetry revealed significant asymmetry *t*(17) = 2.13, *p* = .048. Accordingly, we estimated the effect size after accounting for the small study bias. The limit meta-analyses estimated an effect size of *r* = .01 [95% CI = (-0.05, 0.08)]. A test of small-study effects showed *Q-Q*'(1) = 3.68 *p* = .055 and a test of residual heterogeneity indicated *Q*(17) = 13.81, *p* = .681. Thus, a reanalysis of Bolier et al.’s [23] psychological well-being revealed smaller effect sizes than the effect size of *r* = .10 reported by Bolier et al.

#### Psychological well-being: Complete replication of meta-analysis

Table 4 reports effect sizes determined as described above for each outcome measure and each intervention comparison. These effect sizes were aggregated to yield a single effect size for each study comparable to those reported in Bolier et al. [23]. The correlation between the effect sizes reported by Bolier et al. and the effect sizes calculated through this replication was high, *r* = .88 [95% CI = (0.70, 0.96).

**Table 4:** Effect sizes determined by the current study, for each psychological well-being measure and each study included in Bolier et al. (2013) psychological well-being meta-analysis.

Study	Available Data	Measure	PPI condition	*N*_t_	*N*_c_	*N*_total_	*r*
Abbott.2009.1	prepost-msds	AHI	Reslience program	26	27	53	-.02
Cheavens.2006.1	prepost-msds	TSHS	Hope therapy	16	16	32	.17
Emmons.2003.3	post-anovaF	Connection w/others	Gratitude	33	32	65	.39
Fava.1998.1	prepost-msds	PWB-AU	Well-being therapy	10	10	20	.12
Fava.1998.1	prepost-msds	PWB-EM	Well-being therapy	10	10	20	.20
Fava.1998.1	prepost-msds	PWB-PG	Well-being therapy	10	10	20	.22
Fava.1998.1	prepost-msds	PWB-PR	Well-being therapy	10	10	20	.22
Fava.1998.1	prepost-msds	PWB-PL	Well-being therapy	10	10	20	.01
Fava.1998.1	prepost-msds	PWB-SA	Well-being therapy	10	10	20	.18
Fava.2005.1	prepost-msds	PWB-AU	Well-being therapy	8	8	16	.51
Fava.2005.1	prepost-msds	PWB-EM	Well-being therapy	8	8	16	.54
Fava.2005.1	prepost-msds	PWB-PG	Well-being therapy	8	8	16	.63
Fava.2005.1	prepost-msds	PWB-PR	Well-being therapy	8	8	16	.40
Fava.2005.1	prepost-msds	PWB-PL	Well-being therapy	8	8	16	.62
Fava.2005.1	prepost-msds	PWB-SA	Well-being therapy	8	8	16	.58
Feldman.2012.1	prepost-msds	GSHS-A	Hope-based	32	32	64	-.01
Feldman.2012.1	prepost-msds	GSHS-P	Hope-based	32	32	64	.13
Feldman.2012.1	prepost-msds	PIL	Hope-based	32	32	64	.04
Friesqijk.2006.1	prepost-msds	MAS	Self-management	79	86	165	.06
Gander.2013.1	prepost-msds	AHI	Gratitude visit	61	63	124	.05
Gander.2013.1	prepost-msds	AHI	Three good things	87	63	150	.03
Gander.2013.1	prepost-msds	AHI	Signature strengths	73	63	136	.05
Gander.2013.1	prepost-msds	AHI	Three good things/2 weeks	64	63	127	.12
Gander.2013.1	prepost-msds	AHI	Gratitude & 3 good things	60	63	123	.15
Gander.2013.1	prepost-msds	AHI	Three funny things	55	63	118	-.01
Gander.2013.1	prepost-msds	AHI	Counting kindness	62	63	125	.06
Gander.2013.1	prepost-msds	AHI	Gift of time	55	63	118	-.01
Gander.2013.1	prepost-msds	AHI	One door closes . . .	42	63	105	.03
Green.2006.1	prepost-msds	HTS-C	Solution coaching	25	24	49	.18
Green.2006.1	prepost-msds	PWB-PG	Solution coaching	25	25	50	.13
Green.2006.1	prepost-msds	PWB-EM	Solution coaching	25	25	50	.34
Green.2006.1	prepost-msds	PWB-AU	Solution coaching	25	25	50	.03
Green.2006.1	prepost-msds	PWB-PR	Solution coaching	25	25	50	.35
Green.2006.1	prepost-msds	PWB-PL	Solution coaching	25	25	50	.50
Layous.2013.1	prepost-difmsds	NS-NS	Best possible self	81	38	119	.06
Luthans.2008.1	prepost-msds	PCQ	PsyCap	187	177	364	.05
Luthans.2010.1	prepost-ancovaF	PCQ	PsyCap	153	89	242	.21
Mitchell.2009.1	prepost-msds	OTH-P	Signature strengths	14	23	37	.26
Mitchell.2009.1	prepost-msds	OTH-E	Signature strengths	17	23	40	.18
Mitchell.2009.1	prepost-msds	OTH-M	Signature strengths	17	23	40	-.02
Mongrain.2011.1	prepost-msds	SHIS	Self-compassion	237	237	474	.02
Mongrain.2012.1	prepost-msds	SHI	Positive early memories	87	81	168	-.01
Mongrain.2012.1	prepost-msds	SHI	Three good things	102	81	183	.02
Mongrain.2012.1	prepost-msds	SHI	Signature strengths	74	81	155	.06
Page.2013.1	prepost-msds	PWB	Wellness	23	14	37	.11
Seligman.2006.2	prepost-msds	PPTI	Positive psychotherapy	11	9	20	.40
Spence.2007.1	prepost-msds	PWB-AU	Professional coaching	20	17	37	.40
Spence.2007.1	prepost-msds	PWB-EM	Professional coaching	20	17	37	.13
Spence.2007.1	prepost-msds	PWB-PR	Professional coaching	20	17	37	.07
Spence.2007.1	prepost-msds	PWB-PL	Professional coaching	20	17	37	.35
Spence.2007.1	prepost-msds	PWB-PG	Professional coaching	20	17	37	.35
Spence.2007.1	prepost-msds	PWB-SA	Professional coaching	20	17	37	.28
Spence.2007.1	prepost-msds	PWB-AU	Peer coaching	20	17	37	.28
Spence.2007.1	prepost-msds	PWB-EM	Peer coaching	20	17	37	.14
Spence.2007.1	prepost-msds	PWB-PR	Peer coaching	20	17	37	.12
Spence.2007.1	prepost-msds	PWB-PL	Peer coaching	20	17	37	.43
Spence.2007.1	prepost-msds	PWB-PG	Peer coaching	20	17	37	.30
Spence.2007.1	prepost-msds	PWB-SA	Peer coaching	20	17	37	.33

*Note*. *N*t = treatment sample size; *N*c = control sample size; *N*_total_ = total sample size; prepost-msds = pre and post means and standard deviations; AHI = Authentic Happiness Inventory; TSHS = The State Hope Scale; post-anovaF = anova F statistic from post data only; PWB-AU = Ryff's Psychological Well-Being–Autonomy, PWB-EM = Ryff's Scale of Psychological Well-Being—Environmental mastery; PWB-PG = Ryff's Scale of Psychological Well-Being—Personal growth; PWB-PR = Ryff's Scale of Psychological Well-Being—Positive relations; PWB-PL = Ryff's Scale of Psychological Well-Being—Purpose in life; PWB-SA = Ryff's Scale of Psychological Well-Being–Self-acceptance; GSHS-A = Goal-Specific Hope Scale–agency; GSHS-P = Goal-Specific Hope Scale–pathways; PIL = Purpose in Life Test; MAS = Mastery Scale; HTS-C = Hope Trait Scale composite; NS-NS = Need Satisfaction—not specified; PCQ = Psychological Capital Questionnaire; OTH-P = Orientations to Happiness–pleasure; OTH-E = Orientations to Happiness–engagement; OTH-M = Orientations to Happiness–meaning; SHI = Steen Happiness Index;PWB = Ryff's Psychological Well Being; PPTI = Positive Psychotherapy Inventory

**Fig 15:**
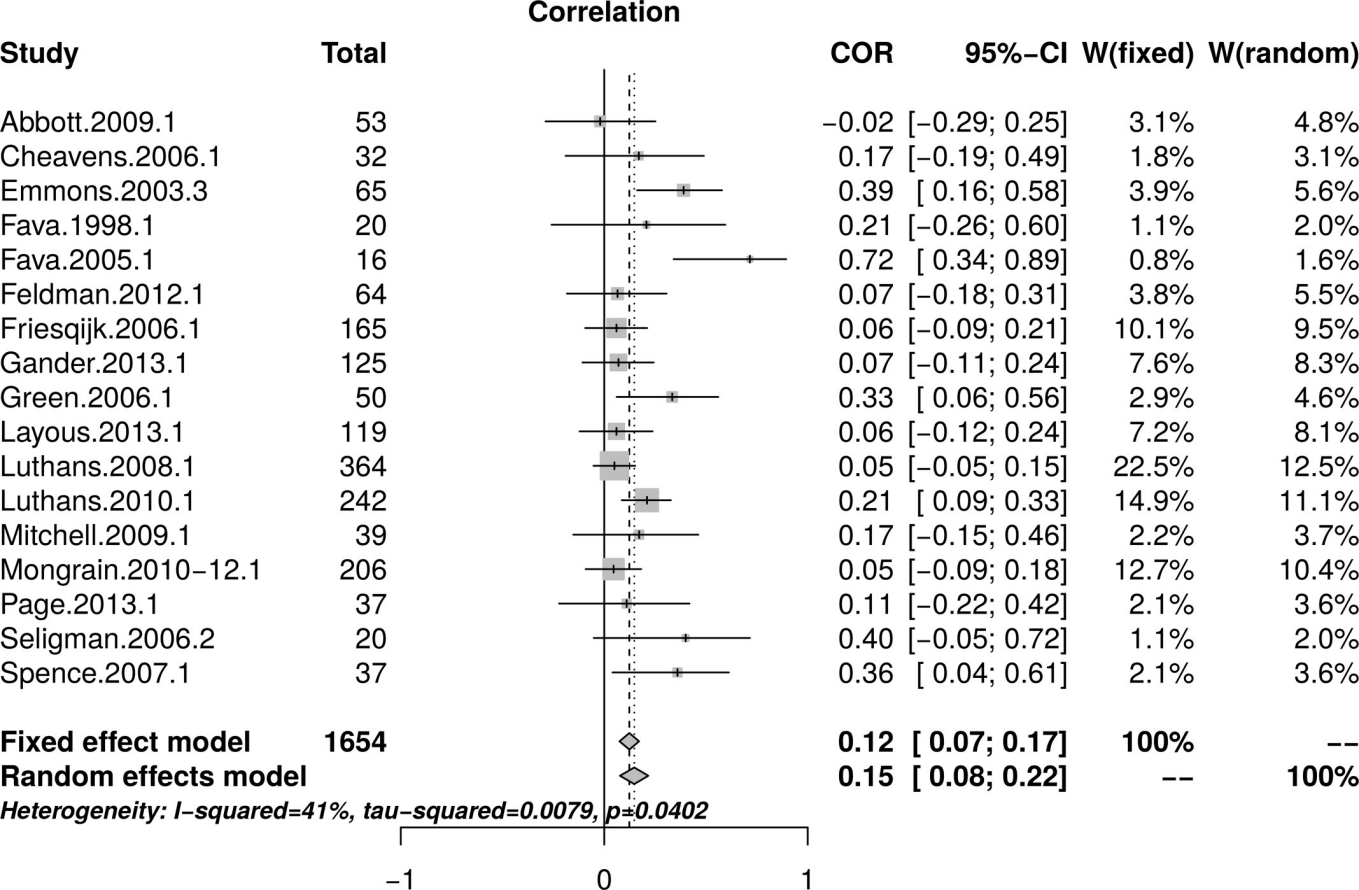
Complete replication of Bolier et al. (2013) psychological well-being effect sizes: Forest plot of study effect sizes. The forest plot indicates that smaller studies reported larger effect sizes than larger studies.

**Fig 16:**
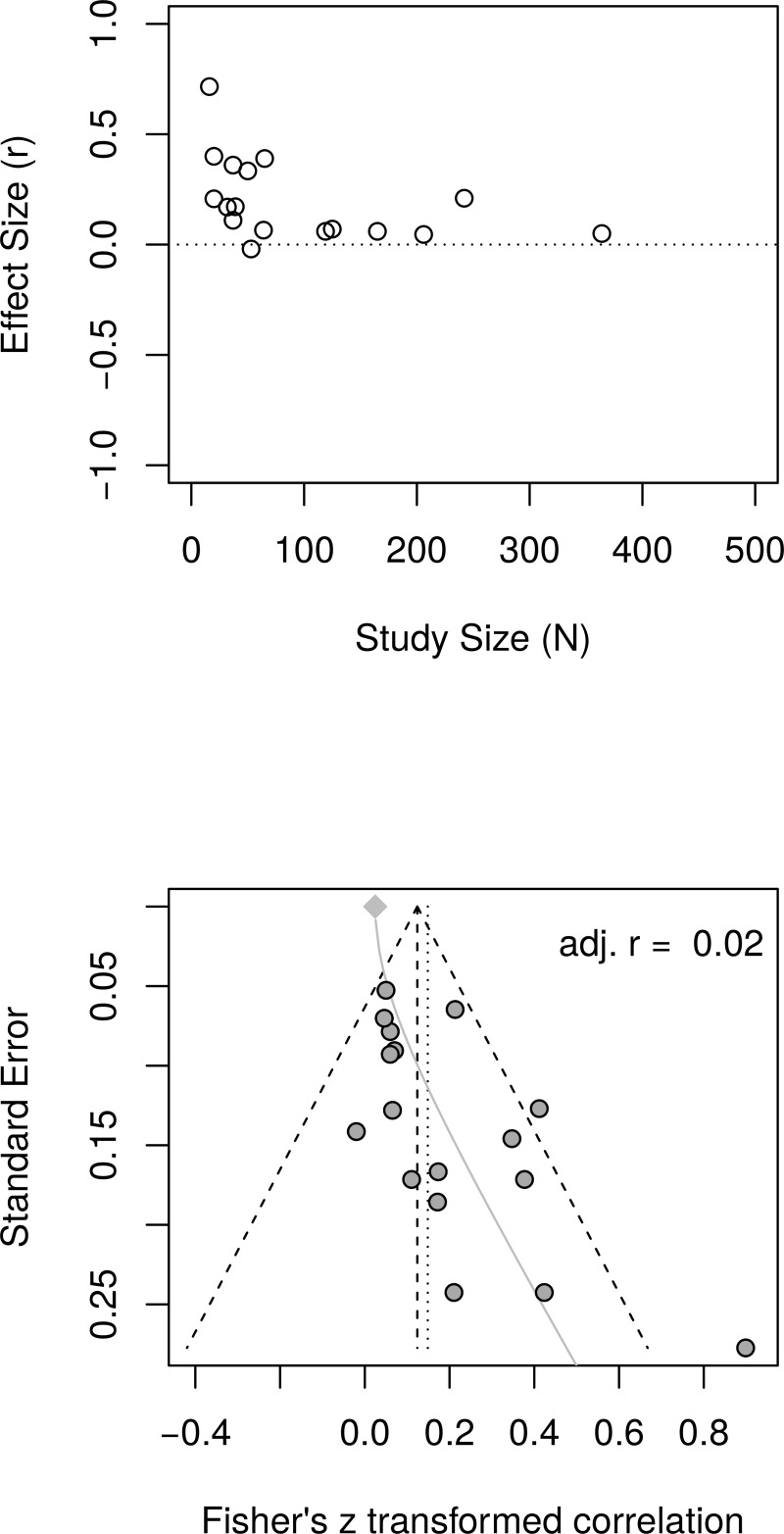
Complete replication of Bolier et al. (2013) psychological well-being effect sizes: Relationship between effect sizes and study sizes. The top panel shows the scatter plot of effect sizes by study sizes. The bottom panel shows the funnel plot and the results of the limit meta-analysis taking into account any small study effects.

Fig 15 shows the forest plot of replication effect sizes. Again, the forest plot indicates that smaller studies reported larger effect sizes than larger studies. A random effect model estimated an effect size of *r* = .15 [95% CI = (0.08, 0.22)] with moderate heterogeneity as measured by *I*^2^, 41.0%. Fig 16, top panel, shows the scatter plot between effect sizes and sample sizes and indicates the presence of small study size bias. Fig 16, bottom panel, shows the funnel plot with visible asymmetry. The regression test of the funnel plot symmetry confirmed the asymmetry, *t*(15) = 2.66, *p* = . 018. Accordingly, it is necessary to estimate the effect size in the presence of the small study size bias. The limit meta-analyses (Fig 16, bottom panel) estimated an effect size of *r* = .02 [95% CI = (-.09, 0.13)]. A test of small-study effects showed *Q-Q*'(1) = 8.71, *p* = .003 and a test of residual heterogeneity indicated *Q*(15) = 18.41, *p* = .242. Thus, a replication of Bolier et al.’s [17] psychological well-being meta-analysis revealed smaller effect sizes than the effect size of *r* = .10 reported by Bolier et al.

#### Depression: Reanalysis of reported data

**Fig 17:**
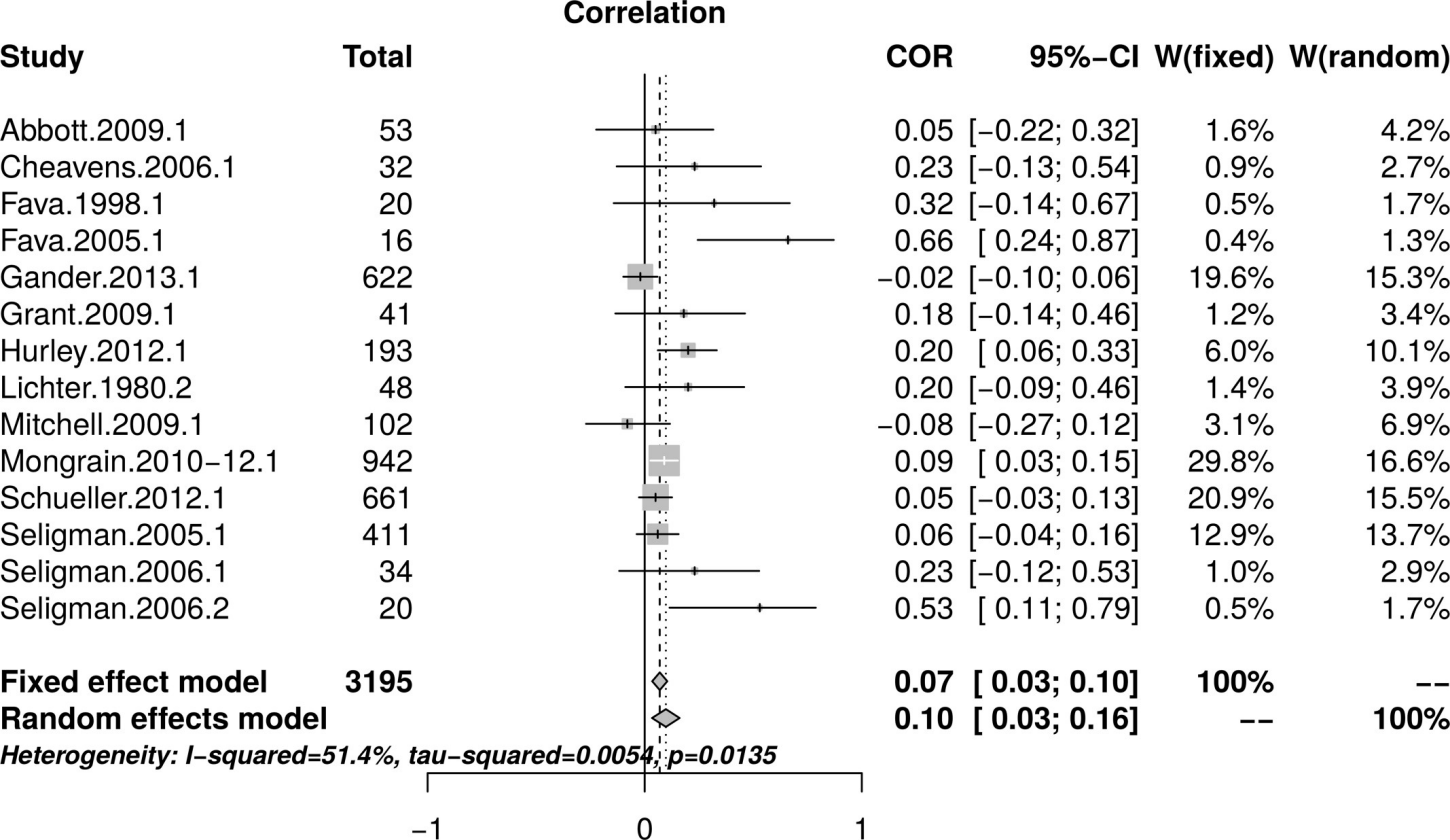
Reanalysis of Bolier et al. (2013) depression effect sizes: Forest plot of study effect sizes. The forest plot indicates that small studies resulted in larger effect sizes than large studies and also suggests the presence of outliers.

A reanalysis used data reported by Bolier et al. [23] in their Table 2 and Fig 4. Fig 17 shows the forest plot of effect sizes. The forest plots indicates that small studies reported larger effect size than larger studies and it also suggests the presence of outliers. A random effect model estimated an effect size of *r* = .10 [95% CI = (0.03, 0.16)] with moderate heterogeneity as measured by *I*^2^ = 51.4%.

**Fig 18:**
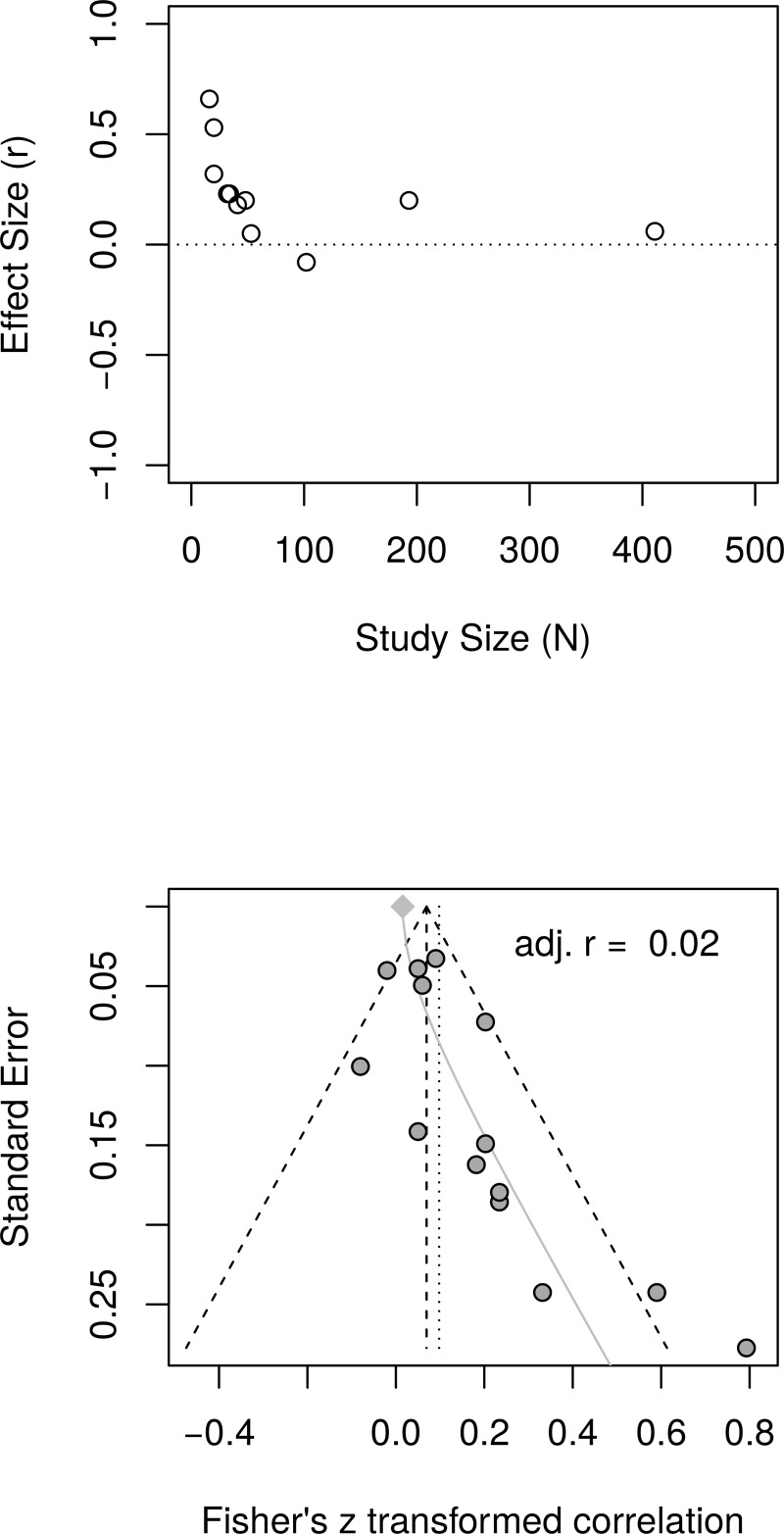
Reanalysis of Bolier et al. (2013) depression effect sizes: Relationship between study sizes and effect sizes. The top panel shows the scatter plot of effect sizes by study sizes. The bottom panel shows the funnel plot and the results of the limit meta-analysis including the estimated effect size taking into account small study effects.

Fig 18, top panel, shows the scatter plot of effect sizes by study size. The scatter plot indicates the presence of small study effects. Fig 18, bottom panel, shows the funnel plot with substantial asymmetry. The regression test of the funnel plot symmetry confirmed the plot was asymmetrical, *t*(12) = 2.71, *p* = .019. Accordingly, it is necessary to estimate the effect size in the presence of the small study size bias. The limit meta-analyses (Fig 18, bottom panel) estimated an effect size of *r* = .02 [95% CI = (-0.04, 0.07)]. A test of small-study effects showed *Q-Q*'(1) = 10.14, *p* = .002 and a test of residual heterogeneity indicated *Q*(12) = 16.60, *p* = .165.

The analyses were repeated after removing the outliers (Fava.2005.1, Seligman.2006.1). A random effect model estimated an effect size of *r* = .07 [95% CI = (0.02, 0.12)] with some heterogeneity as measured by *I*^2^ = 27.7%. The regression test of the funnel plot symmetry revealed no significant asymmetry, *t*(10) = 1.55, *p* = .152. The limit meta-analyses estimated an effect size of *r* = .03 [95% CI = (-0.03, 0.10)]. A test of small-study effects showed *Q-Q*'(1) = 2.95, *p* = .086 and a test of residual heterogeneity indicated *Q*(10) = 12.27, *p* = .268. Thus, the reanalyses of Bolier et al.’s data revealed a smaller, non-significant effect for depression, in contrast to Bolier et al.’s finding of *r* = .11.

#### Depression: Complete replication of meta-analysis

**Fig 19:**
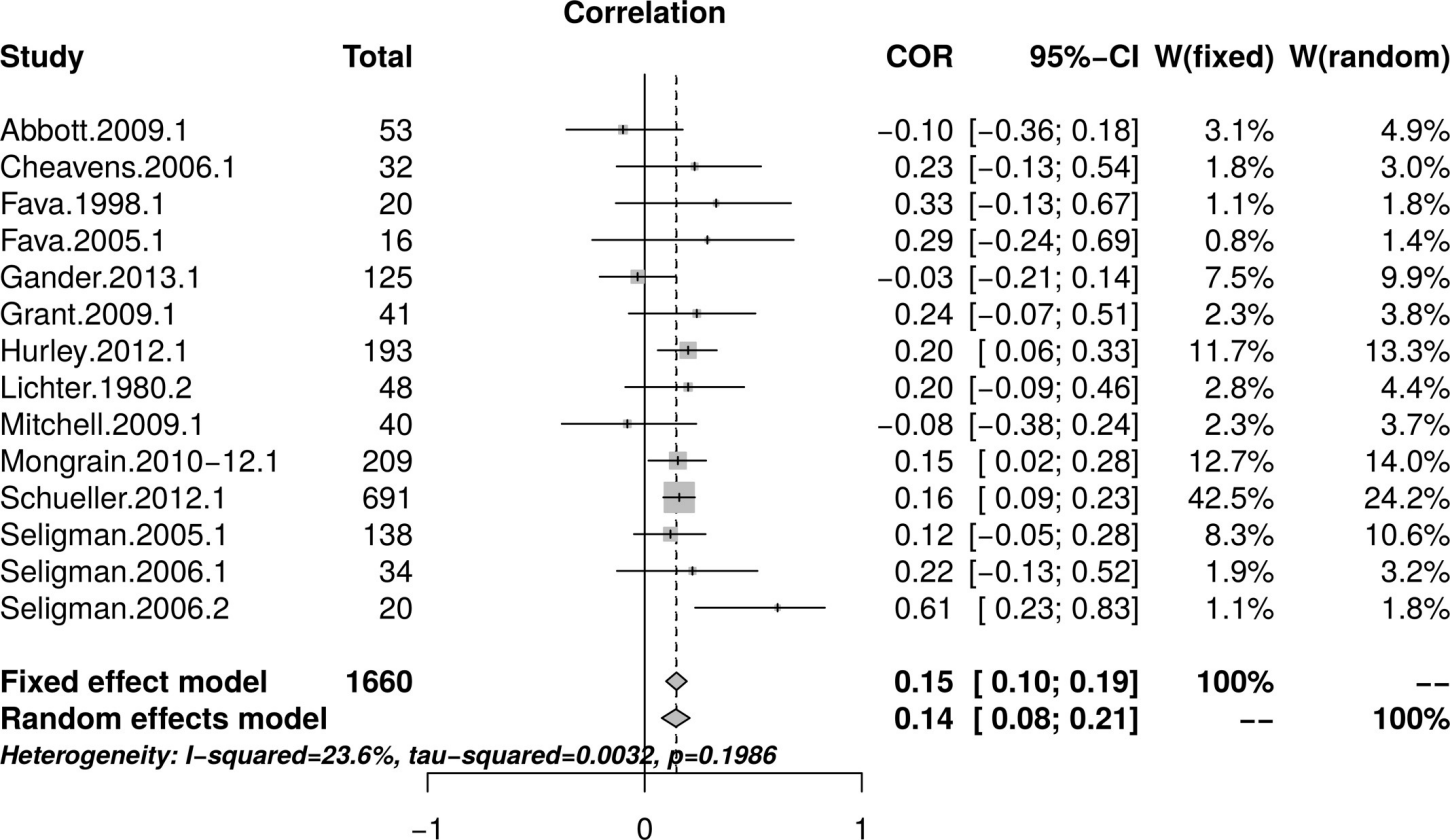
Complete replication of Bolier et al. (2013) depression effect sizes: Forest plot of effect sizes. The forest plot shows some scatter and suggests the presence of an outlier.

Table 5 reports effect sizes determined as described above for each outcome measure and each intervention comparison. These effect sizes were aggregated to yield a single effect size for each study comparable to those reported in Bolier et al. [23]. The correlation between the effect sizes reported by Bolier et al. [23] and the effect sizes calculated through this replication was high, *r* = .81 [95% CI = (0.49, 0.94). Fig 19 shows the forest plot of effect sizes and displays no apparent small study size effects. A random effect model estimated an effect size of *r* = .14 [95% CI = (0.08, 0.21)] with moderate heterogeneity as measured by *I*^2^ = 23.6%.

**Table 5:** Effect sizes determined by the current study, for each depression measure and each study included in Bolier et al. (2013) depression meta-analysis.

Study	Available Data	Measure	PPI condition	*N*t	*N*c	*N*.total	*r*
Abbott.2009.1	prepost-msds	DASS-D	Reslience program (online)	26	27	53	-.10
Cheavens.2006.1	prepost-msds	CES-D	Hope therapy	16	16	32	.23
Fava.1998.1	prepost-msds	CID-DEP	Well-being therapy	10	10	20	.53
Fava.1998.1	prepost-msds	SQ-DEP	Well-being therapy	10	10	20	.04
Fava.2005.1	prepost-msds	CID-DEP	Well-being therapy	8	8	16	.28
Fava.2005.1	prepost-msds	SQ-DEP	Well-being therapy	8	8	16	.22
Gander.2013.1	prepost-msds	CES-D	Gratitude visit	61	63	124	-.10
Gander.2013.1	prepost-msds	CES-D	Three good things	87	63	150	-.01
Gander.2013.1	prepost-msds	CES-D	Signature strengths in a new way	73	63	136	-.06
Gander.2013.1	prepost-msds	CES-D	Three good things in the last two weeks	64	63	127	.00
Gander.2013.1	prepost-msds	CES-D	Gratitude visit & three good things	60	63	123	.10
Gander.2013.1	prepost-msds	CES-D	Three funny things	55	63	118	-.06
Gander.2013.1	prepost-msds	CES-D	Counting kindness	62	63	125	-.03
Gander.2013.1	prepost-msds	CES-D	Gift of time	55	63	118	-.10
Gander.2013.1	prepost-msds	CES-D	One door closes, another opens	42	63	105	.04
Grant.2009.1	prepost-msds	DASS-D	Leadership development program	20	21	41	.24
Hurley.2012.1	prepost-msds	BDI-II	Savouring the moment	94	99	193	.20
Lichter.1980.2	prepost-msds	BDI	Positive feeling statements	25	23	48	.20
Mitchell.2009.1	prepost-msds	DASS-D	Signature strengths in a new way	17	23	40	-.08
Mongrain.2011.1	prepost-msds	CES-D	Self-compassion	237	237	474	.15
Mongrain.2012.1	prepost-msds	CES-D	Positive early memories	90	84	174	.06
Mongrain.2012.1	prepost-msds	CES-D	Three good things	106	84	190	.12
Mongrain.2012.1	prepost-msds	CES-D	Signature strengths in a new way	75	84	159	.14
Schueller.2012.1	prepost-msds	CES-D	Three good things & signature strengths	326	355	681	.16
Schueller.2012.1	prepost-msds	CES-D	Three good things & signature strengths & gratitude visit & savouring	364	355	719	.18
Schueller.2012.1	prepost-msds	CES-D	Three good things & signature strengths & gratitude visit & savouring & active-constructing respondng & life summary	319	355	674	.05
Seligman.2005.1	prepost-msds	CES-D	Gratitude visit	80	70	150	.16
Seligman.2005.1	prepost-msds	CES-D	Three good things	59	70	129	.10
Seligman.2005.1	prepost-msds	CES-D	You at your best	68	70	138	.10
Seligman.2005.1	prepost-msds	CES-D	Signature strengths in a new way	66	70	136	.07
Seligman.2005.1	prepost-msds	CES-D	Identifying signature strengths	68	70	138	.03
Seligman.2006.1	prepost-msds	BDI-II	Positive psychotherapy	14	20	34	.22
Seligman.2006.2	prepost-msds	ZSRS	Positive psychotherapy	11	9	20	.47
Seligman.2006.2	post-msds	HRSD	Positive psychotherapy	11	9	20	.59
Sergeant.2011.1	prepost-nomsnosds	CES-D	Gratitude	NA	NA	NA	NA
Shapira.2010.1	prepost-msds	CES-D	Self-compassion	63	70	133	.06
Shapira.2010.1	prepost-msds	CES-D	Optimism	55	70	125	.17

*Note*. *N*t = treatment sample size; *N*c = control sample size; *N*_total_ = total sample size; prepost-msds = pre and post means and standard deviations; DASS-D = Depression Anxiety Stress Scale–Depression; CES-D = Center for Epidemiologic Studies Depression Scale; CID-DEP = Clinical Interview for Depression; SQ-DEP = Kellner's Symptom Questionnaire–Depression; BDI-II = Beck Depression Inventory-II; BDI = Beck Depression Inventory; post-msds = post means and standard deviations only; ZSRS = Zung Self-Rating Scale for Depression; HRSD = Hamilton Rating Scale for Depression; prepost-nomsnods = pre and post no means and no standard deviations

**Fig 20:**
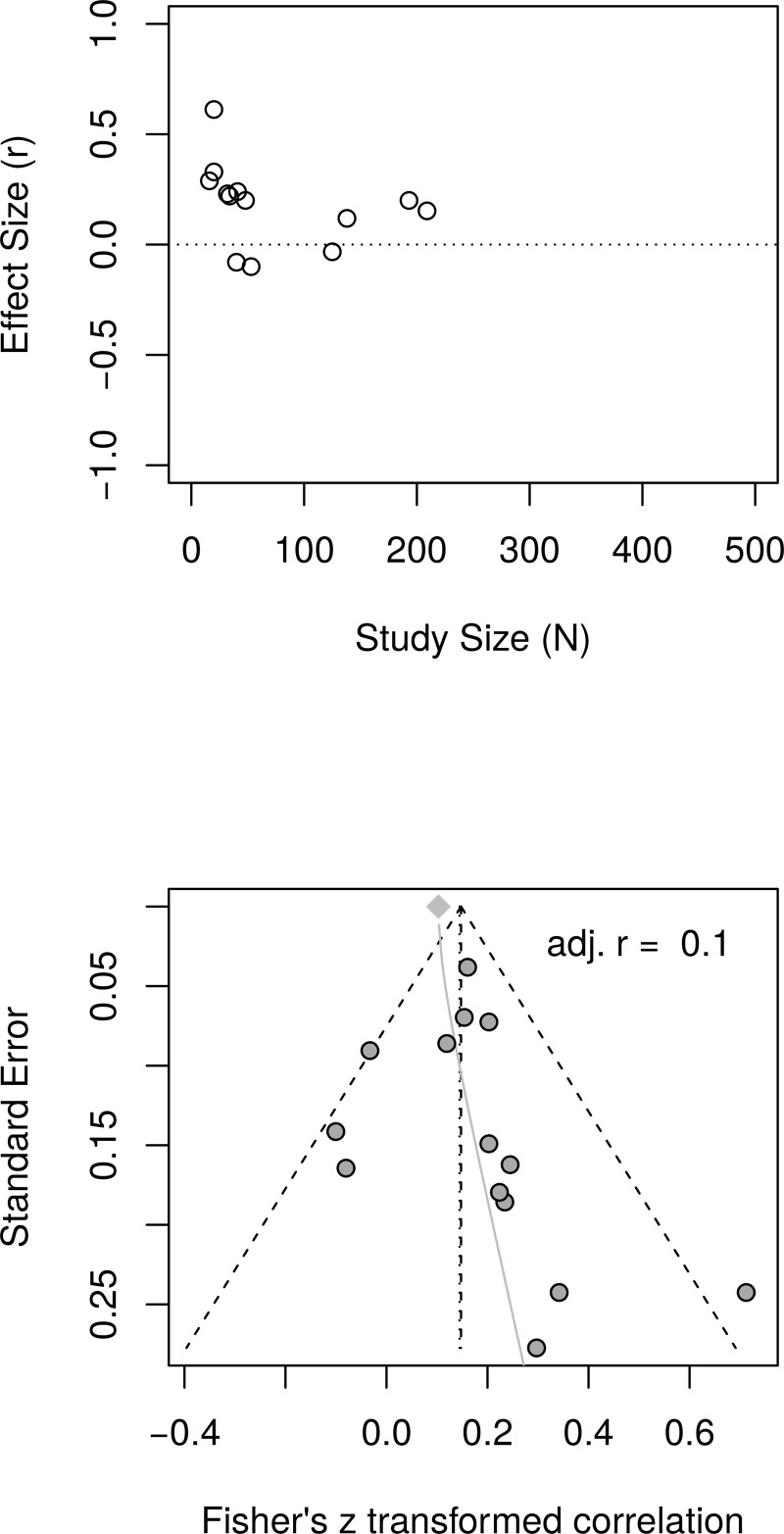
Complete replication of Bolier et al. (2013) depression effect sizes: Relationship between effect sizes and sample sizes. The top panel shows the scatter plot of effect sizes by study sizes. The bottom panel shows the funnel plot and the results of the limit meta-analysis including the estimated effect size taking into account small study effects.

Fig 20, top panel, shows the scatter plot of effect sizes and sample sizes. Smaller studies tend to show larger effects than large studies. Fig 20, bottom panel, shows the funnel plot with some asymmetry. However, a regression test of the funnel plot symmetry indicated no statistically significant asymmetry, *t*(12) = .52, *p* = .611. The limit meta-analyses (Fig 20, bottom panel) estimated an effect size of *r* = .10 [95% CI = (.01, 0.19)]. A test of small-study effects showed *Q-Q'*(1) = 0.38, *p* = .539 and a test of residual heterogeneity indicated *Q*(12) = 16.64, *p* = .164. However, these results are difficult to interpret due to the small number of studies.

The effect size estimates were recalculated after the removal of an outlier (Seligman.2006.2). A random effect model estimated an effect size of *r* = . 14 [95% CI = (.09, .19)] with no heterogeneity as measured by *I*^2^ = 0%. A regression test of the funnel plot symmetry indicated no statistically significant asymmetry, *t*(11) = -.17, *p* = .862. The limit meta-analyses estimated an effect size of *r* = .15 [95% CI = (.06, 0.24)]. A test of small-study effects showed *Q-Q'*(1) = .03, *p* = .862 and a test of residual heterogeneity indicated *Q*(11) = 11.51, *p* = .402. The replication analyses indicated a somewhat higher effect for depression than that reported by Bolier et al. [23].

### Summary

Table 6 summarizes the key findings from our reanalyses of Sin and Lyubomirsky and Bolier et al. meta-analyses. For comparison, it also includes effect sizes (*r*s) originally reported by Sin and Lyubomirsky and Bolier et al. The table highlights that re-analyses of the data reported in the two previous meta-analyses resulted in much smaller effect sizes than those originally reported. Moreover, of the seven meta-analyses that yielded significant findings in the previously conducted studies, only two remained statistically significant and one more depended on one outlier when reanalyzed in the current study.

**Table 6:** Summary of reanalyses of the previous meta-analyses.

		Original Analyses	Reanalyses			
	*k*	*r* (95% C.I.)	RE *r* (95% C.I.)	FAT (*p)*	LMT *r* (95% C.I.)	LMT *d*
**Sin & Lyubomirsky (2009)**						
Well-being	49	**.29 (.21, .37)**	**.24 (.18, .30)**	**< .001**	**.08 (.00, .15)**	**.16**
Depression	25	**.31 (.17, .43)**	**.25 (.14, .34)**	**.004**	.04 (-.05, .13)	.08
**Bolier et al. (2013)**						
Subjective Well-being	28	**.17**	**.17 (.11, .22)**	.299	**.13 (.02, .24)**	**.26**
Psychological Well-being	20	**.10**	**.09 (.04, .14)**	**.015**	.02 (-.04, .08)	.04
Psychological Well-being (w/o outliers)		**.08**	**.06 (.03, .10)**	**.048**	.01 (-.05, .08)	.02
Depression	14	**.11**	**.10 (.03, .16)**	**.019**	.02 (-.04, .07)	.04
Depression (w/o outliers)	12	**.09**	**.07 (.02, .12)**	.152	.03 (-.03, .10)	.06

*Note*. Bold print = *p* < .05; boldface = significant findings; RE *r* = random effects model estimate of *r*; FAT (*p*) = Funnel plots of asymmetry *p* value; LMT *r* = Limit Meta-analysis effect size estimate of *r*; LMT *d* = Limit Meta-analysis effect size in *d*

Table 7 summarizes the key findings from our complete replications of Sin and Lyubomirsky and Bolier et al. meta-analyses. The table highlights that our replications showed generally small effects of PPI on well-being and depression that were comparable to the effects found by our re-analyses of Sin and Lyubomirsky and Bolier et al.’s data.

**Table 7:** Summary of replications of the previous meta-analyses.

	*k*	RE *r* (95% C.I.)	FAT (*p)*	LMT *r* (95% C.I.)	LMT *d*
**Sin & Lyubomirsky (2009)**					
Well-being	40	**.23 (.17, .30)**	**.003**	.10 (-.01, .20)	.20
Depression	21	**.26 (.14, .38)**	**< .001**	-.03 (-.17, .11)	.06
**Bolier et al. (2013)**					
Subjective Well-being	25	**.19 (.12, .26)**	.184	**.13 (.00, .26)**	.26
Psychological Well-being	17	**.15 (.08, .22)**	**.018**	.02 (-.09, .13)	.04
Depression	14	**.14 (.08, .21)**	.611	**.10 (.01, .19)**	**.20**
Depression (w/o outliers)	13	**.14 (.09, .19)**	.868	**.15 (.06, .24)**	**.30**

*Note*. Bold print = *p* < .05; boldface = significant findings; RE *r* = random effects model estimate of *r*; FAT (*p*) = Funnel plots of asymmetry *p* value; LMT *r* = Limit Meta-analysis effect size estimate of *r*; LMT *d* = Limit Meta-analysis effect size estimate in *d*

## Discussion

The first meta-analysis examining the effectiveness of the PPIs on well-being, by Sin and Lyubomirsky [22], reported moderate effects on improving well-being and decreasing depression. A second meta-analysis by Bolier et al. [23] focused on randomized trials only and found much smaller effects of PPIs than the first meta-analysis. Bolier et al. attributed their smaller effects to their inclusion of higher quality studies only. However, in addition to the differences in the inclusion criteria, our detailed reading of the two meta-analyses suggested an alternative explanation for the discrepancy in the reported effect sizes. The discrepancy may be due to common methodological issues affecting many published meta-analyses including (a) the failure to weigh studies by their sample size, (b) the failure to describe the calculation of effect sizes in sufficient detail, and (c) the failure to consider and adjust for small sample size bias. Therefore, though Schueller et al. [24] correctly criticized Bolier et al. study because of the unreasonably narrow selection criteria and cautioned against drawing any conclusions from Bolier et al. meta-analysis, there may be additional reasons that warrant caution.

Accordingly, our study had two major objectives. First, we reanalyzed the reported data from the two previous meta-analyses while taking into account study sizes and small sample size bias. Second, we replicated both meta-analyses starting with extracting relevant effect sizes directly from the primary studies rather than relying on the data published in the previous meta-analyses. In conducting these meta-analyses, the data were analyzed using a weighted random effects model while taking into account small sample size bias using the selected methods discussed above.

Our reanalysis of the effect sizes reported by Sin and Lyubomirsky [22] revealed much smaller effect size estimates for both well-being (*r* = .08) and depression (*r* = .04) than the previous authors reported (*r* = .29 and *r* = .31, respectively). There were two major reasons for the inflated estimates reported by Sin and Lyubomirsky. First, Sin and Lyubomirsky reported effect size estimates as simple unweighted averages of study level effect sizes (i.e., they averaged *r*s across the studies included in their meta-analysis). This approach is inappropriate because it gives equal weight to small- and large-size studies [26]. Second, Sin and Lyubomirsky noted that their effect sizes resulted in asymmetric funnel plots, but they used F*ail Safe N* to conclude that small-study effects did not significantly inflate their findings. However, the F*ail Safe N* is no longer considered an appropriate way to assess small-study effects [26]. The present study’s reanalysis confirmed that the funnel plots were asymmetric for both well-being and depression, and the random effects limit meta-analysis estimates are much smaller (and not statistically significant for depression) due to small-study effects. The replication of Sin and Lyubomirsky [22] meta-analyses revealed relatively high correlations between effect sizes determined by the current study and by those in the previous study for both well-being and depression. Consistent with the similar effect sizes extracted from the primary studies, the replication analyses and estimated effect sizes for well-being and for depression were very similar to those obtained by our reanalyses of effect sizes reported by Sin and Lyubomirsky. The replication analyses resulted in nearly the same findings as those from the reanalyses even though several studies that did not report essential data to calculate effect sizes were excluded from the replications.

Our reanalysis of the effect sizes reported by Bolier et al. [23] revealed the same estimated effect size for subjective well-being (*r* = .17) as reported by Bolier et al. However, the estimated effect sizes for psychological well-being (*r* = .02), and depression (*r* = .02) were smaller (and no longer statistically significant) than originally reported in Bolier et al. (*r* = .10, and *r* = .11, respectively). When outliers were removed, the estimated effect sizes for psychological well-being were *r* = .01 and for depression were *r* = .07. The latter result is partially attributable to the test of funnel plot asymmetry being no longer statistically significant, in part due to the smaller number of effect sizes included. However, the limit meta-analysis estimated the effect size for depression after the removal of outliers as *r* = .03. The replication of Bolier et al. [23] meta-analyses revealed relatively high correlations between effect sizes determined by the current study and those reported in their meta-analysis for subjective well-being, psychological well-being, and depression. Despite the removal of several original studies (due to insufficient data to calculate effect sizes), the results of the replication analyses of subjective well-being and psychological well-being were very similar to those obtained by the reanalyses. The replication of depression effects resulted in slightly larger estimated effect sizes of *r* = .14. However, these results need to be viewed with caution as they are based on a small number of studies. Moreover, even though the small-study effects were not statistically significant, the number of studies was small and the scatter plots of effect sizes and study sample sizes show that large-size studies resulted in substantially smaller effects than small size studies.

In summary, the reanalyses and replications of Sin and Lyubomirsky [22] and Bolier et al. [23] indicate that there is a small effect of approximately *r* = .10 of PPIs on well-being. In contrast, the effect of PPIs on depression was nearly zero when based on the studies included in Sin and Lyubomirsky [22] and highly variable, and sensitive to outliers, when based on studies included in Bolier et al. [23]. Notably, Sin and Lyubomirsky [22] included nearly twice as many studies as Bolier et al. [23] in their meta-analysis of the effects of PPIs on depression.

Our review of the two highly cited meta-analyses of PPIs resulted in a number of secondary findings and implications. First, the major reason for the larger effects reported in previous meta-analyses was that these studies did not appropriately account for prevalent small-study effects. The small-study effects are a frequent problem with meta-analyses in many fields and a number of methods (e.g., cumulative meta-analysis, TOP10, limit meta-analysis) have been developed to estimate effect sizes in the presence of small-study effects. Unfortunately, these methods were not employed in the previous meta-analyses addressed by the current study. Given the presence of the small-study effects, future meta-analyses of PPIs must take into account small-study effects using appropriate estimation methods.

Second, these findings are tentative because the previous meta-analyses did not include all available studies. To illustrate, Bolier et al.’s [23] inclusion criteria are restrictive because they excluded (a) all relevant studies published prior to the coining of the term “Positive Psychology”, (b) all studies of effects of mindfulness and meditation on well-being, and (c) all studies that did not explicitly mention “positive psychology”. As pointed out by Schueller, et al. [24], Bolier et al.’s inclusion criteria are too narrow and exclude numerous studies that use the same interventions and same outcome measures. If a substantial number of relevant studies were not included, the findings based on only a small sample of relevant studies may not reflect the cumulative findings across the population of previous studies. In turn, not conducting a comprehensive search for primary studies also reduces meta-analysists’ ability to conduct meaningful moderator analyses [24].

Third, the failure to include all available studies in the previous meta-analyses suggests the need for a comprehensive meta-analysis of PPIs effect on well-being starting with a comprehensive search for relevant studies. A preliminary search using PsycInfo for studies of PPIs using only the most obvious search strategy (search for all studies mentioning both “positive psychology” and at least one of the terms “intervention”, “therapy”, or “treatment”) yielded over 200 relevant studies, more than tripling the number of studies included in the previous meta-analyses.

Fourth, our review of the primary studies included in the previous meta-analyses revealed persistent limitations with their method and results sections. In general, no primary studies with pre-post designs reported pre-post correlations for outcome measures, which are necessary to calculate the most appropriate effect sizes [101]. Though the authors of a number of these primary studies were contacted by email, they did not provide these correlations. As a result, the current study relied primarily on the post data only, following the approach adopted by Bolier et al. [23]. Accordingly, these findings suggest that researchers need to report all necessary statistical information to facilitate future replication and/or meta-analyses. Although numerous guidelines have been provided for reporting the results of studies such as JARS [124], researchers appear slow to adopt them, and the present findings suggest the need to push for adoption of such guidelines by researchers in the PPI field.

Fifth, it is evident from the diverse inclusion and exclusion criteria of previous meta-analyses that there is no consensus as to what constitutes a PPI. Bolier et al. [23] excluded interventions that others consider PPIs (e.g., mindfulness and meditation). Bolier et al. even speculated that different inclusion criteria and differences in study designs were the reason for discrepancies between their findings and those of Sin and Lyubomirsky [22]. However, the current reanalysis casts doubt on this explanation as the findings were comparable when small-study effects were taken into account. The definition of a PPI is critical for determining which studies to include in future meta-analyses. Schueller et al. [24] argued that including only studies that mention “positive psychology” would miss many 'positive intervention' studies. Similarly, Parks and Biswas-Diener [125] acknowledged that it can be arduous to define interventions that are aimed at increasing the ‘positives’. Clearly, this needs to be addressed in the near future.

Thus, the “true” effects of PPIs may be substantially different from what Sin and Lyubomirsky and Bolier et al. meta-analyses indicate. While our re-analyses and replications of these meta-analyses converge and indicate that the effect of the PPIs are relatively small when small sample bias is taken into account, estimates of effect sizes are not definitive because neither Sin and Lyubomirsky nor Bolier et al. meta-analyses were comprehensive and a large number of relevant studies are likely missing.

Accordingly, a comprehensive and transparent meta-analysis of all relevant studies of PPIs is necessary and is likely to have a major influence on the field. Such a meta-analysis is likely to allow for meaningful moderator analyses in answering questions such as: Is group administration more effective than individual administration? Are longer interventions more effective than shorter interventions? Are some types of interventions more effective than other types of interventions? Importantly, a comprehensive meta-analysis is likely to provide a more definitive determination of how effective PPIs are at increasing well-being.

Given that our meta-analyses indicate that the effects of the PPIs on well-being and depression may be smaller than previously reported, future research may need to employ strategies likely to increase the effectiveness of PPIs. For example, PPIs are likely to be more effective if they are deployed over longer periods of time [126]. Some researchers have criticized the use of single short duration PPIs in some areas [127] and others have argued that PPIs ought to be deployed over longer periods of times [128] as was done in only some of the PPI studies [129]. Moreover, the effectiveness of the PPIs may depend not only on overall duration but also on frequency of PPIs. Finally, it may be that a combination of two or three PPIs (e.g., the combination of best possible self and gratitude letters) is more effective than a single type of PPI of equal duration [130].

## Conclusions

The current study re-analyzed the data reported in previous meta-analyses that examined the effectiveness of PPIs on increasing well-being and decreasing depression, as well as completely replicated (extracting data from original sources) the previous meta-analyses. The reanalysis of the previously reported data showed that although correlations between the recalculated effect sizes and the previous meta-analyses effect sizes were fairly high (suggesting that the same data were extracted), the effect sizes were lower than previously reported and often nonsignificant. The major contributing factor for this discrepancy was that the present study accounted for the strong presence of small-sample size bias. Critically, both meta-analyses reviewed, did not include a large number of relevant studies, and thus, effect sizes estimated from their sample of primary studies need to be confirmed by future, more comprehensive, meta-analyses. Accordingly, a comprehensive and transparent meta-analysis of all relevant studies of PPIs is necessary. Such a meta-analysis will allow for meaningful moderator analyses to determine effects of various PPIs including whether individual PPIs are more effective than group PPIs and whether longer and more intense PPIs are more effective than shorter and less intense interventions. Our research underscores that any future meta-analyses of PPI effectiveness ought to take into account frequent methodological issues such as prevalent small sample size bias.
